# Antisense Oligonucleotides as a Gene-Silencing Strategy Regulating Cytosolic G6PDH in *Hordeum vulgare*

**DOI:** 10.3390/plants15142223

**Published:** 2026-07-21

**Authors:** Antonella Aquilone, Maryanna Martina Perrotta, Simone Landi, Sergio Esposito

**Affiliations:** 1 Dipartimento di Biologia, University of Napoli Federico II, Via Cinthia, 80126 Napoli, Italy; a.aquilone@ssmeridionale.it (A.A.); maryannamartinaperrotta@gmail.com (M.M.P.); sergio.esposito@unina.it (S.E.); 2 Scuola Superiore Meridionale, Largo San Marcellino 10, 80138 Naples, Italy; 3 Department of Chemical Sciences, University of Napoli Federico II, 80126 Naples, Italy

**Keywords:** ASOs, barley, oligo technology, ROS, redox, HSP70, shaggy kinase

## Abstract

Antisense oligonucleotides (ASOs) are short, synthetic DNA fragments able to modulate gene expression. In this work, the cytosolic isoform of glucose-6-phosphate dehydrogenase (Cyt-G6PDH) was selected as a target for ASOs to induce transient gene silencing in barley (*Hordeum vulgare*). G6PDH is the most important enzyme of the oxidative pentose phosphate pathway (OPPP), supplying NADPH and regulating the entire cycle. Different ASOs were tested at different concentrations on the leaf surface. Their effects were evaluated by measuring enzymatic activity, gene expression, and protein abundance. Treatment with 30 µM ASOs for 6 h represented the optimal condition, inducing a 40–60% reduction in G6PDH total activity in barley leaves, with a specific decrease in the cytosolic isoform, as determined by DTT-sensitive enzymatic assays. ASOs designed on the main regulator of cyt-G6PDH, the shaggy-like kinase (SK11), led to an analogous effect in terms of G6PDH activity, confirming the involvement of HvSK11 in the regulation of cyt-G6PDH in barley. Consistently, qRT-PCR analyses showed that ASO treatment simulates an abiotic stress condition obtained by the down-regulation of Cyt-G6PDH. These results support the use of ASOs as a rapid and efficient method for the functional analysis of key metabolic regulators in plants to overcome complications in recalcitrant organisms or lethal genes.

## 1. Introduction

Gene silencing is a powerful molecular strategy to inhibit and regulate the expression of specific targets [1,2,3,4]. Plants naturally employ gene silencing to defend cells against aberrant RNA molecules that can cause damage and disrupt cellular functions [5]. There are two main gene silencing mechanisms in plants: transcriptional gene silencing (TGS) and post-transcriptional gene silencing (PTGS) [2,6]. TGS works in the nucleus, inhibiting transcription by preventing the binding of the transcriptional machinery to gene promoters [7]. PTGS is a cytoplasm-localized mechanism that precisely targets and degrades mRNA transcripts expressed by specific genes [7]. Common PTGS approaches include RNA interference (RNAi), antisense RNA (asRNA), clustered regularly interspaced short palindromic repeats (CRISPR/Cas9), and different forms of non-coding RNAs (ncRNAs) [7,8]. Among these, RNAi has been extensively used to study gene function, improve pest resistance, and investigate plant developmental and physiological processes [9]. RNAi involves small non-coding RNAs, such as small interfering RNAs (siRNAs) and microRNAs (miRNAs), which are generated as cleavage products from double-stranded RNA (dsRNA) [10]. This cleavage process is mediated by ribonucleases known as DICER or DICER-like enzymes (DCLs) [11]. In addition, RNAi relies on the activity of the RNA-induced silencing complex (RISC), which incorporates the small RNAs to guide gene silencing and Argonaute (AGO) proteins, which execute the cleavage or translational repression of target mRNAs [12,13,14,15]. By contrast, antisense RNA technology involves RNA molecules modified to create a complementary copy of mRNA, which regulates the expression of the coding protein [4,16,17].

ASOs are short, synthetic DNA molecules that bind sequence-specifically to target mRNAs, leading to the translational arrest or RNase H-mediated degradation of the transcript [18]. The use of ASO introduced into cells to modulate gene expression is an emerging, alternative strategy for specific purposes in plant studies, representing a tool that opens unique possibilities for gene control [19]. This strategy was originally developed for therapeutic applications in mammals, but recently, plant biology research has become interested in ASO as a versatile tool for transient gene silencing [18,19,20]. ASO technology is a low-cost and easily implemented method for the direct manipulation of gene activity. The main advantage of this method is that gene expression can be modified with no requirement for stable transformation, avoiding any long-term or lethal effects on plants [21,22]. Oligo technology is not commonly used in plants compared with animals but has already been shown to work in several species, namely *Nicotiana Tabacum* or *Camellia sinensis* [23,24].

This technology was used in plant cells to alter the expression of a gene, encoding the transcription factor SUSIBA2, thus demonstrating that ASO could efficiently be transported from the nucleus to chloroplasts in leaves [25]. The presence of the cell wall makes plant cells more sensitive to ASO treatments compared with animal cells. The cell wall is more permeable to negatively charged oligonucleotide molecules [19]. Additionally, ASOs can enter plant cells through specialized channels for sugar molecules [25]. Different methods were designed to transfer ASOs into plant cells, including reduced-pressure infiltration, infiltration through stomata, cell spraying, forced osmosis, and a biological particle delivery system [19,26].

In this work, we investigated the use of ASOs as a transient gene-silencing strategy. We selected the cytosolic isoform of glucose-6-phosphate dehydrogenase, G6PDH (EC 1.1.1.49), as a target, the main and regulatory enzyme of the oxidative pentose phosphate pathway (OPPP) [27]. G6PDH catalyzes the oxidation of glucose-6-phosphate (G6P) to 6-phospho-δ-glucono-1,5-lactone. Several studies have recently described the critical function of G6PDH in the response to abiotic stress in plants. When subjected to abiotic stress, G6PDH counteracts the oxidative burst, acting as the main non-photosynthetic NADPH supplier [28,29,30,31]. This cofactor will then be used by scavenging enzymes involved in the detoxification of reactive oxygen species (ROS) [31].

The presence of at least four different G6PDH isoforms was demonstrated in higher plants [32]. Two compartmentalized enzymes can be found in plastids: P1-G6PDH in chloroplasts and P2-G6PDH in non-photosynthetic plastids [33]. An alternative isoform, P0-G6PDH, which lacks catalytic activity, was reported as stress-responsive, contributing to the activation of an additional OPPP into peroxisomes [34,35].

The major contribution (60–80%) in total G6PDH activity can be ascribed to the cytosolic isoform Cyt-G6PDH [27]. This isoform is regulated by different factors, including abscisic acid (ABA) [36], nitrogen [28], and abiotic stresses [31,37]. It is worth pointing out that Cyt-G6PDH is insensitive to light, while this factor mainly controls the activity of P1-G6PDH [38,39]. In *Arabidopsis thaliana*, Cyt-G6PDH (G6PD6—At5g40760) is activated by the shaggy-related kinase 11 (AtSK11—AT5G26751). Shaggy-like kinases, also known as glycogen synthase kinases (GSKs), are highly conserved serine/threonine kinases present in both animals and plants [40]. These threonine/serine kinases are expressed both in the nucleus and cytoplasm. In the nucleus, GSKs act as transcriptional inhibitors of target gene expression [40]. In the cytoplasm, GSKs regulate various proteins through phosphorylation. Additionally, GSK3s also regulate post-translational modifications of proteins, including acetylation, phosphorylation, and oxidative status [40]. Plant genomes contain multiple isoforms of GSK, which are involved in various biological processes [40,41,42,43].

Although shaggy-like kinases are best known as negative regulators of brassinosteroid (BR) signalling, they also participate in the crosstalk of other signalling pathways, including abscisic acid (ABA), salinity, cold stress, and light perception [40]. Through the phosphorylation of downstream substrates, SKs coordinate stress-responsive gene expression, cellular redox homeostasis, and metabolic adaptation, thereby balancing plant growth and stress responses [40,43]. Members of the shaggy kinase family act as regulators of transcription factors by the phosphorylation and degradation of these [43]. This mechanism in *A. thaliana* regulates various physiological processes and responses to environmental stimuli. For example, BIN2 (AT4G18710, AtSK21) regulates the degradation of ICE1 (inducer of CBF expression 1), influencing the transcriptional cascades activated by plants subjected to low temperature [40]. Furthermore, shaggy kinases have been shown to directly modulate the activity of metabolic enzymes, including cyt-G6PDH. In this case, AtSK11 enhances the enzymatic activity of G6PDH, contributing to ROS homeostasis during salinity stress [40].

This study aimed to investigate the use of ASOs for the transient silencing of Cyt-G6PDH and of the regulator SK11. We selected barley (*Hordeum vulgare*) as the model species. Unlike the classic model species *Arabidopsis thaliana*, barley is a genetically recalcitrant crop exhibiting low transformation efficiencies due to limited in vitro regeneration capacity and strong genotype dependence [44,45]. In addition, to our knowledge, no studies about the silencing of Cyt-G6PDH and about the regulation by SK11 were published on barley.

## 2. Results

### 2.1. HvG6PDH Alignment and Antisense Oligonucleotides Design

The barley (*Hordeum vulgare*) genome revealed the presence of three genes coding for cyt-G6PDH, namely MLOC_13014, MLOC_7841, and MLOC_10808 [31]. These isoforms showed different expression patterns in various tissues, with MLOC_7841 and MLOC_10808 being the most highly expressed cytosolic isoforms [28]. To develop effective ASOs to regulate Cyt-G6PDH enzymatic activity by transient silencing, two different ASOs were designed based on sequence alignment. First, the genomic sequences of MLOC_13014, MLOC_7841, and MLOC_10808 were aligned with their respective CDSs (Supplementary Figure S1) to avoid overlaps at exon/intron junctions by selecting the 3′ portion of the mRNA. Subsequently, the CDSs of MLOC_13014, MLOC_7841, and MLOC_10808 were realigned (Supplementary Figure S2) to confirm the presence of the selected ASO sequences in each transcript. The resulting ASOs were named *HvASOcytA* and *HvASOcytB*.

### 2.2. Antisense Oligonucleotide Strategy Is a Good Approach to Manage the Activity of Cyt-G6PDH

**Figure 1:**
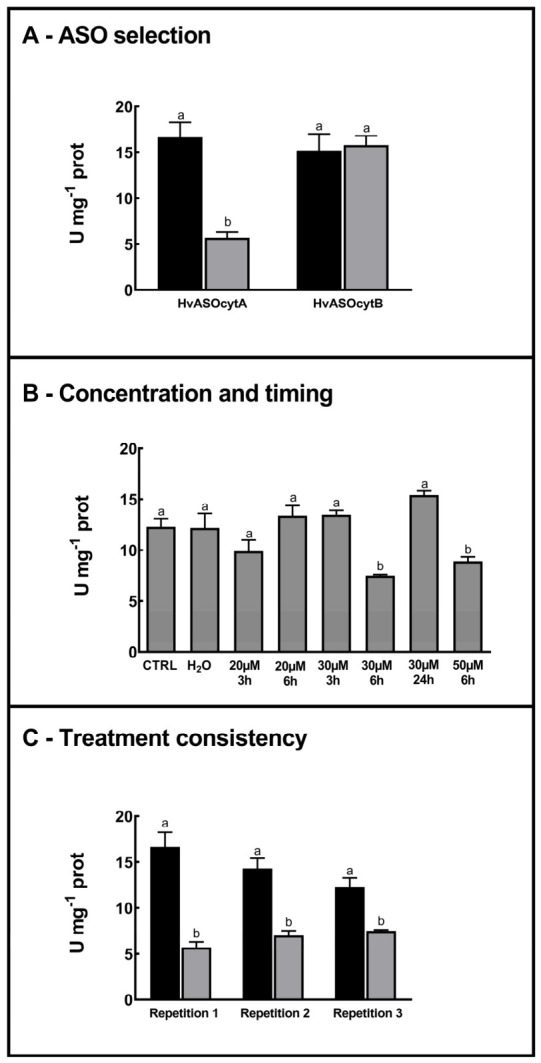
(**A**) Total enzymatic activity of G6PDH in leaves of barley plants CTRL (black bars) and treated with different ASOs (*HvASOcytA* and *HvASOcytB*—grey bars). (**B**) Total enzymatic activity of G6PDH in barley plants CTRL, treated with H_2_O, and treated with *HvASOcytA* at different concentrations for 3 h, 6 h, and 24 h. (**C**) Total enzymatic activity of G6PDH in leaves of CTRL plants (black bars) and treated with *HvASOcytA* at 30 µM for 6 h (grey bars) from different levels of hydroponic growth. Letters indicate the statistical significance between CTRL and treated plants using ANOVA.

Different experimental strategies were employed to establish an effective protocol for the silencing of barley Cyt-G6PDH via ASO administration. Experiments using *HvASOcytA* resulted in a specific reduction in total G6PDH activity in treated leaves (−66%), whereas leaves from untreated control (CTRL) plants and *HvASOcytB*-treated plants showed no significant differences (Figure 1A). Based on these results, HvASOcytA was selected for further analysis, while *HvASOcytB* was excluded from subsequent experiments.

Several conditions were analyzed to verify the appropriate *HvASOcytA* concentration, exposure time, and treatment duration. Specifically, concentrations of 20 µM, 30 µM, and 50 µM were assessed for 3 h, 6 h, and 24 h (Figure 1B). *HvASOcytA*-treated leaves were compared to untreated barley leaf samples (CTRL) and water-treated barley leaf samples. The results showed that 20µM *HvASOcytA* did not significantly reduce G6PDH total activity. Furthermore, it was also observed that 3 h of exposure to *HvASOcytA* was not sufficient to cause effects at the different concentrations used. Interestingly, 30 µM *HvASOcytA* for 6 h induced a significant reduction (−40%) in total G6PDH activity. *HvASOcytA* effects ended after 24 h of exposure. Similarly, samples treated with 50 µM *HvASOcytA* for 6 h showed a reduction in total G6PDH activity (−25%).

Notably, no significant differences were observed between the controls and the water-treated leaves, confirming that changes in G6PDH activity were specifically caused by *HvASOcytA* treatment, rather than mechanical damage or wounding stress.

Treatment with 30 µM *HvASOcytA* for 6 h was re-proposed in hydroponically grown barley plants (Figure 1C). Each biological replicate was made by growing untreated control (CTRL) and treated plants separately in a hydroponic system. These analyses confirmed the effects of *HvASOcytA* treatment. Control leaves showed similar levels of G6PDH activity from 16.6 to 12.3 U mg^−1 prot^. By contrast, *HvASOcytA*-treated leaves exhibited a significant reduction from −40 to −65%. To evaluate species-specificity, *Arabidopsis thaliana* plants were exposed to the treatment by using two different ASOs: the barley-functioning *HvASOcytA* and a specific ASO designed cytosolic G6DPH from *A. thaliana* (*AtASOcytA*). As shown in Supplementary Figure S3, the barley-specific ASO caused no effects in *A.thaliana*, while *AtASOcytA* induced a significant reduction in G6PDH activity (−50%).

### 2.3. Specificity of HvASOcytA vs. Cyt-G6PDH

**Figure 2:**
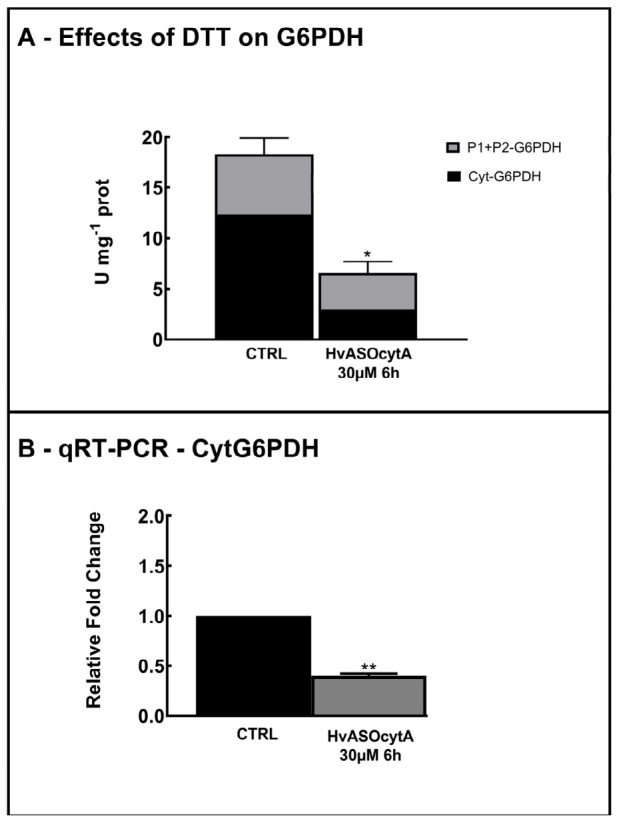
(**A**) Enzymatic activity contribution of cytosolic isoform (black bars) and compartmentalized isoforms (P1 + P2—grey bars) in CTRL and *HvASOcytA* at 30 µM for 6 h. (**B**) Changes in the gene expression of Cyt-G6PDH in barley plants of CTRL (black bar) and treated with *HvASOcytA* 30 µM for 6 h (grey bar) by qRT-PCR. mRNA levels were calculated relative to the expression of the α-tubulin used as calibrator. Expression values were reported as -fc (fold change—ratio of the normalized expression value—ASO treated vs. CTRL). Asterisks indicate significant differences between treated and control plants (* = *p* ≤ 0.05; ** = *p* ≤ 0.005).

To investigate the effect of *HvASOcytA* to specifically manipulate the activity of Cyt-G6PDH, two different approaches were used: DTT assay and qRT-PCR (Figure 2). DTT-sensitive activity assays allowed discrimination between Cyt-G6PDH and plastidial isoforms based on the susceptibility of P1/P2-G6PDH to reducing agents [46]. As shown in Figure 2A, *HvASOcytA* determined a specific reduction in Cyt-G6PDH activity in treated leaves. Total G6PDH activity was allocated as 74% for Cyt-G6PDH and 36% for plastidial enzymes (P1/P2-G6PDH) in control plants. Conversely, *HvASOcytA*-treated plants showed an altered proportion of the different isoforms; Cyt-G6PDH contributed 45% of the total activity, while the compartmentalized isoforms contributed 54%. Consistently, expression analyses focused on Cyt-G6PDH showed a down-regulation (-2.5-fold change) in plants treated with *HvASOcytA* (Figure 2B).

### 2.4. Different Strategies to Regulate Cyt-G6PDH by ASO: The Role of SK11

Cyt-G6PDH is regulated through phosphorylation by SK11 kinase, which plays a key role in the positive regulation of this enzyme [47]. Therefore, we designed two different ASOs (*HvASOsk11A* and *HvASOsk11B*) to specifically silence SK11. The barley genome reported different putative candidates as HvSK11 (HORVU3Hr1G034440; HORVU3Hr1G034440 and HORVU1Hr1G016490) [48]. The phylogenetic tree showed that HvGSK1.3 (HORVU1Hr1G016490) clustered together with AtSK11 and AtSK12 in a specific sub-branch. Therefore, this specific sequence was selected to design ASOs (Supplementary Figure S4).

**Figure 3:**
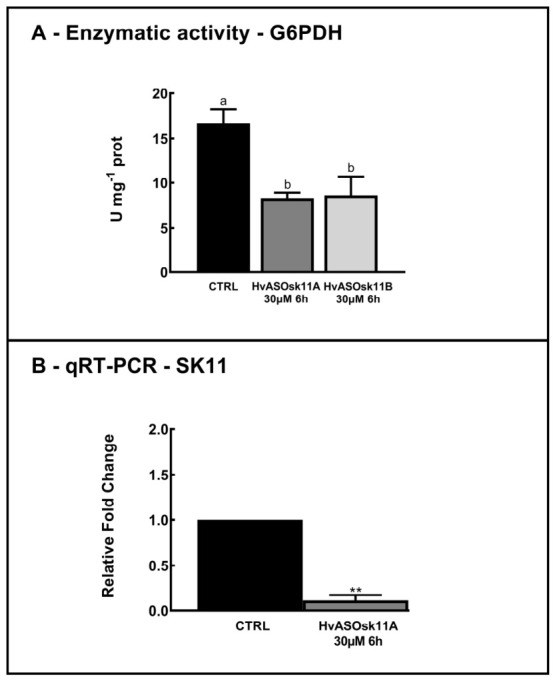
(**A**) Total enzymatic activity of G6PDH in leaves of barley plants CTRL (black bar) and treated with 30 µM of HvASOsk11A (dark grey bar) and HvASOsk11B (light grey bar) for 6 h. Letters indicate the statistical significance between CTRL and treated plants using ANOVA. (**B**) Changes in the gene expression of SK11 in barley plants of CTRL (black bar) and treated with HvASOsk11A 30 µM for 6 h (grey bar) by qRT-PCR. mRNA levels were calculated relative to the expression of the α-tubulin used as calibrator. Expression values were reported as -fc (fold change—ratio of the normalized expression value—ASO treated vs. CTRL). Asterisks indicate significant differences between treated and control plants (** = *p* ≤ 0.005).

Barley leaves were exposed to the same conditions defined before (30 µM for 6 h). As shown in Figure 3, treatment with both *HvASOsk11A* and *HvASOsk11B* resulted in a significant reduction in total G6PDH activity (Figure 3A). It was observed that the total G6PDH activity in controls remained stable at 16.2 ± 1.2 U mg^−1 prot^, while exposure to *HvASOsk11A* and *HvASOsk11B* induced a significant reduction in total G6PDH activity, −50.2% and −48.3%, respectively.

These results are related to the specific reduction in SK11 expression in barley leaves treated with *HvASOsk11A*, as shown by qRT-PCR analyses, indicating a severe reduction in SK11 gene expression (−9.4 fc) compared to control plants. (Figure 3B).

**Figure 4:**
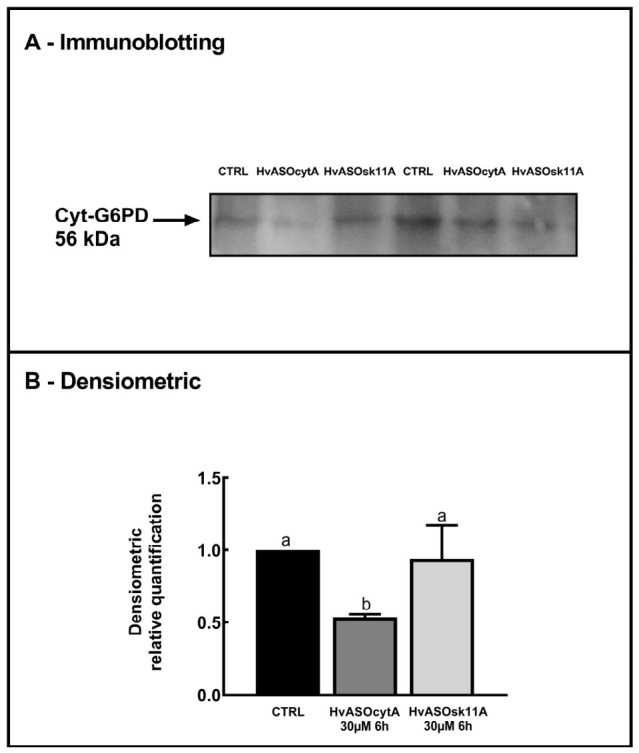
Immunoblotting (**A**) using antisera for Cyt-G6PDH (56 kDa) and densiometric quantification (**B**) of leaf extracts from barley plants under CTRL conditions (black bar) and in plants treated with *HvASOcytA* (dark grey bar) and *HvASOsk11A* (light grey bar) at 30 µM for 6 h. Letters indicate the statistical significance between CTRL and treated plants using ANOVA.

Moreover, we tested the presence of Cyt-G6PDH—in extracts of barley leaves treated with *HvASOcytA* and *HvASOsk11A*—by immunoblotting, using specific antibodies (Figure 4). Equal protein loading was evaluated through Comassie blue incubation (Supplementary Figure S5).

This analysis revealed a reduction in Cyt-G6PDH levels in plants treated with *HvASOcytA*. Intriguingly, leaves treated with *HvASOsk11A* reported no or reduced effects on Cyt-G6PDH presence, confirming a regulation at the post-transcriptional level.

### 2.5. Effects of HvASOcytA and HvASOsk11A in the Regulation of 6-Phosphogluconate Dehydrogenase

**Figure 5:**
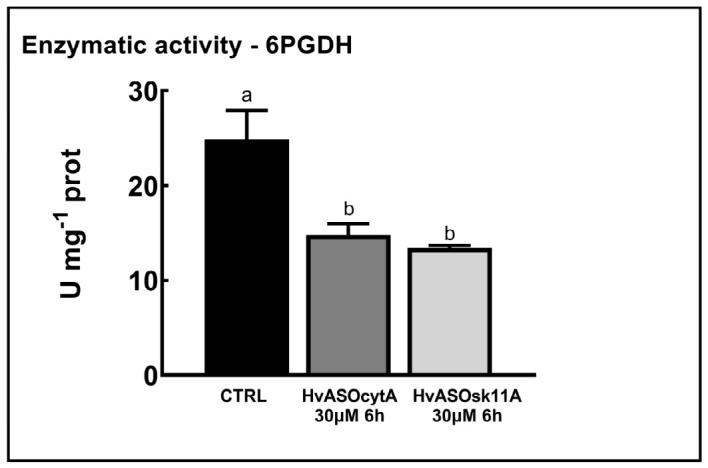
Total enzymatic activity of 6PGDH in leaves of barley plants CTRL (black bar) and treated with 30 µM of *HvASOcytA* (dark grey bar) and *HvASOsk11A* (light grey bar) for 6 h. Letters indicate the statistical significance between CTRL and treated plants using ANOVA.

**Figure 6:**
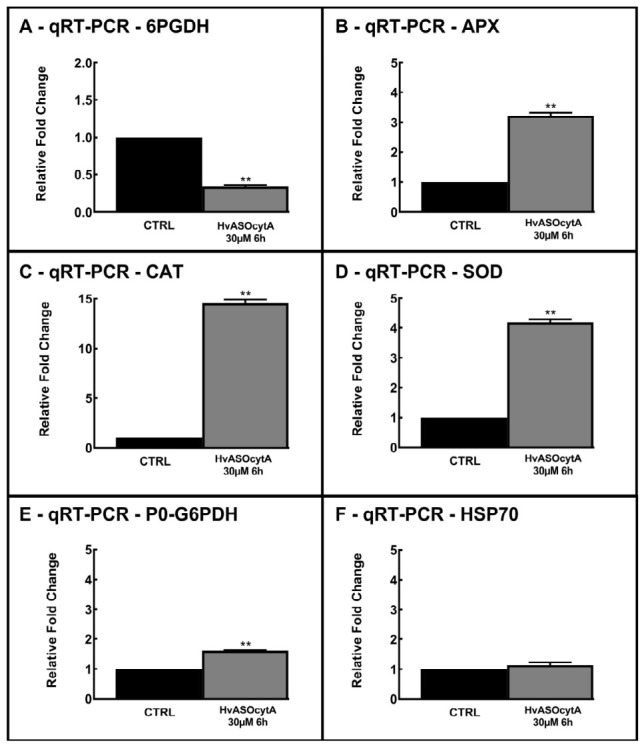
Changes in the gene expression of 6PGDH (**A**), APX (**B**), CAT (**C**), SOD (**D**), P0-G6PDH (**E**), and HSP70 (**F**), in barley plants CTRL (black bars) and treated with *HvASOcytA* 30 µM for 6 h (grey bars). mRNA levels were calculated relative to the expression of the α-tubulin used as calibrator. Expression values were reported as -fc (fold change—ratio of the normalized expression value—ASO-treated vs. CTRL). Asterisks ** = indicate significant differences between treated and control plants (*p* ≤ 0.005).

The ASO-dependent regulation of G6PDH activity suggested that the whole oxidative phase of OPPP could be, at least in part, regulated by the same mechanism. Therefore, 6-phosphogluconate dehydrogenase (6PGDH) activity was measured in barley plants treated with 30 µM *HvASOcytA* and *HvASOsk11A* for 6 h (Figure 5). The results showed an effective regulation of 6PGDH as well. Barley leaves showed a similar reduction in total 6PGDH enzymatic activity when treated with both *HvASOcytA* (−40.5%) and *HvASOsk11A* (−46%). Consistently, qRT-PCR confirmed the down-regulation (-2.9-fc) of 6PGDH expression in HvASOcytA-treated plants (Figure 6A).

### 2.6. Molecular Effects of Cyt-G6PDH Silencing in Barley Plants

Cyt-G6PDH is a well-known abiotic stress-responsive gene in barley [49]. To investigate the consequences of the use of ASO in silenced plants, leaves of barley plants treated with 30 µM *HvASOcytA* for 6 h were analyzed by qRT-PCR (Figure 6). Specific genes involved in the response to oxidative stress, namely ascorbate peroxidase (APX), catalase (CAT), superoxide dismutase (SOD), P0-G6PDH, and Heat Shock protein 70 (HSP70), were selected.

As shown in Figure 6B–D, APX, CAT, and SOD genes showed a significant up-regulation in treated barley leaves, about +3.2 fc, +14.5 fc, and +4.2 fc, respectively. Interestingly, the expression of P0-G6PDH, the enzymatically inactive G6PDH isoform responsible for the activation of an additional stress-responsive peroxisomal OPPP, showed a +1.6 fc increase in gene expression (Figure 6E). By contrast, the gene encoding the cytosolic HSP70 showed no significant difference in gene expression comparing controls and *HvASOcytA*-treated leaves (Figure 6F).

## 3. Discussion

Gene silencing by antisense oligonucleotides (ASOs) is an emerging approach in plant biotechnology, allowing the down-regulation of target genes through RNA hybridization. In plants, this mechanism plays an essential role in developmental processes, genome stability, and defence against viruses [19,50]. Modifications induced by ASOs can replicate phenotypic traits observed in reference transgenic plants, without permanent or deadly changes and overcoming technical challenges [19,51].

In the current study, we selected silencing by ASOs as a strategy to modulate the expression of an important enzyme of primary plant metabolism: cyt-G6PDH. As a model organism, we selected barley, one of the most cultivated crops in the world [52]. To our knowledge, no previous attempts were made in the use of ASOs targeted to critical enzymes involved in barley central metabolism. Different species have been utilized to investigate the effects of ASOs, including tobacco (*Nicotiana benthamiana*), wheat (*Triticum aestivum*), *Citrus sinensis*, and *A. thaliana* [21,23].

Previously, it has been reported that the use of ASOs can obtain transient gene silencing in the nuclear-encoded phytoene desaturase (pds), the chlorophyll a/b binding protein (cab) genes, and the chloroplast-encoded psbA gene [23]. ASOs were modified by the addition of phosphorothioate to improve oligonucleotide stability. Gene silencing could be obtained by incubating ASOs on seeds or on excised leaf tissue [53]. These strategies induced the temporary but severe inhibition of seed germination and leaf growth, lasting about 10 days [53]. In contrast, our experimental strategy was designed by applying ASOs directly onto the leaf surface without any chemical modification of the oligo.

ASO delivery is a crucial factor determining the success of gene silencing in plants. Unlike animal cells, plant cells have additional physical barriers, including the cuticle and cell wall, which limit the uptake of exogenous nucleic acids [20,21,22]. However, several studies have shown that exogenously administered oligonucleotides can be transported into plant tissues and reach intracellular compartments [20,23,25]. Different delivery methods have been developed for introducing oligonucleotides into plant cells, including infiltration under reduced pressure, stomatal infiltration, foliar spraying, and absorption via sugar solutions [7,19,21,22,24]. Each approach has specific advantages and limitations, depending on species, tissue, developmental stage, and experimental objective.

In the current study, we intentionally selected a simple topical application by manual brushing to evaluate whether naked ASOs were sufficient to induce transient silencing of Cyt-G6PDH and to ensure the homogeneous distribution of the ASO solution and prolonged contact with leaf surfaces, thereby maximizing the opportunity for foliar absorption while minimizing solution loss. Although intracellular ASO uptake was not directly quantified, the significant reduction in Cyt-G6PDH activity observed after *HvASOcytA* treatment demonstrates that the applied ASOs reached their intracellular target at biologically relevant levels.

Our data clearly show that treatment with ASOs designed on Cyt-G6PDH allowed an efficient reduction in the enzymatic function, indicating the effectiveness of this silencing strategy in barley. Interestingly, only one designed ASO (*HvASOcytA*) was effective in the silencing of Cyt-G6PDH isoforms. This difference in behaviour could be due to small differences in the ASO and Cyt-G6PDH CDS sequences (Supplementary Figure S2). The most efficient treatment conditions were achieved using a concentration of 30 µM ASO for 6 h, causing a reduction in total G6PDH activity ranging from 40% to 60%. ASO stability and absorption represent critical issues in the use of this technology. In different studies using ASOs for plant-silencing approaches, it was necessary to manipulate ASOs (by protection or by diluting them into sugar solution) to improve both stability and absorption [19,21,25]. However, different authors agree that the effectiveness of ASOs could be dependent on many aspects, such as plant species, tissues, and developmental stage [21].

Similar results to those obtained with *HvASOcytA* were obtained by silencing different G6PDH isoforms using a conventional method in *A. thaliana* (T-DNA insertion) [54,55,56] or an innovative strategy, CRISPR-CAS9, in *Zea mays* [57]. Interestingly, although cyt-G6PDH sequences are highly conserved across different species, experiments using ASOs designed for barley and *A. thaliana* have demonstrated high species-specificity in silencing. ASOs designed for barley showed no activity in modulating *At*G6PDH activity. In any case, this finding requires further investigation, including studies on different enzymes and more closely related species.

The successful regulation of Cyt-G6PDH by ASO in barley was confirmed through qRT-PCR, immunoblotting, and enzymatic assays in the presence of dithiothreitol (DTT), a reducing agent that selectively inhibits the chloroplastic/plastidic isoforms (P1/P2-G6PDH) but not the cytosolic form [46]. The use of ASO reframes the contribution of the different isoforms to the total activity of G6PDH. Untreated plants showed the classical contribution: 70–80% (Cyt-G6PDH); 30–20% (P1/P2-G6PDH) [27]. Plants treated with ASO shifted to 45% (Cyt-G6PDH) and 54% (compartmentalized isoforms).

Similar reorganization in G6PDH contribution in total activity was reported in a g6pd5/g6pd6 knock-out genotype [58] and in the *Arabidopsis thaliana* mutant lacking g6pd1 [54]. Interestingly, the inhibition of neither the cytosolic isoform nor the chloroplastic isoform is deadly. A lack of cytosolic isoforms induced significant phenotypic damage to root growth, silique development, seed germination and embryo development, clearly due to the prominent role that cytosolic G6PDH plays in plant cell metabolism [55,56]. The absence of the chloroplastic isoform induced modifications in lipid and fatty acid metabolism and chloroplast development [54]. More generally, the alteration of G6PDH isoforms severely affects nitrogen assimilation in monocotyledon crops [58].

Interestingly, treatment with ASO targeted on Cyt-G6PDH also allows us to regulate the whole OPPP, considering that 6PGDH was also affected by the treatment. This result highlights the entire OPPP slowing down and the strict connection between the two dehydrogenases of the pathway. These results also confirm the critical role of G6PDH as a key regulator of the entire OPP pathway [32].

The effects of ASO at the molecular level were evaluated by checking the expression of critical genes involved in the oxidative stress response: APX, CAT, and SOD. These genes exhibited a significant up-regulation in treated barley leaves. These results suggest that the use of ASO on Cyt-G6PDH simulates an oxidative stress condition. Cyt-G6PDH is an important enzyme for the response of plants to different abiotic stresses (e.g., drought and low temperature) acting in the replenishment of reductants required by scavenging enzymes [28,36,49]. Abiotic stresses increase ROS levels [59,60,61] and the expression and the activities of G6PDH, together with scavenging enzymes as well as APX, CAT, SOD, and others [31,49,60,62]. The activity of Cyt-G6PDH is often related to NAPDH content [31,54]. The up-regulation reported in the ASO-treated plants of APX, CAT, and SOD could therefore be related to an increased requirement of NAPDH by these enzymes. It is worth pointing out that P0-G6PDH is up-regulated in ASO-treated barley plants. This result reinforces the activation of the abiotic stress response again. This isoform is involved in the translocation of P1/P2-G6PDH to peroxisomes to improve the oxidative stress response [34,35]. It is worth remembering that the regulation or depletion of G6PDH expression and activity (in silenced or KO genotypes) is related to the modification in ROS levels and/or NADP^+^/NADPH ratio [31,54,57,58]. Therefore, it is reasonable to suggest that the silencing of Cyt-G6PDH by ASO could similarly regulate these aspects of plant metabolism.

Consistently, the effects of cyt-G6PDH deficiency were studied in maize, highlighting a defective response to low temperature, observing significant modifications in the redox status of ascorbic acid, glutathione, and NADPH [57]. In contrast, the expression of cytosolic HSP70, one of the most important chaperones and marker of abiotic stress in plants [63], did not significantly differ between control and ASO-treated plants. This suggests that the silencing of Cyt-G6PDH did not induce severe and prolonged stress conditions in leaf cells enough to require protein protection by chaperones but only an increased need for NADPH (not required by HSP70).

We also evaluated the possible regulation by phosphorylation of Cyt-G6PDH, designing an ASO targeted to the kinase SK11 [47]. In *A. thaliana*, the regulation of Cyt-G6PDH activity is achieved by a specific shaggy-related kinase, AtSK11 (AT5G26751). This enzyme plays a post-translational role in the regulation of Cyt-G6PDH, phosphorylating Thr-467 and increasing the enzymatic activity of the enzyme [47]. The expression of AtSK11 is up-regulated upon salinity [47] and low temperatures [54], improving the activity of Cyt-G6PDH under these stress conditions.

*A. thaliana* plants defective for SK11 showed salt-stress susceptibility by reduced germination and root growth [47]. Our results clearly showed a significant reduction in total G6PDH activity (approximately 50%) in leaves treated with ASO, which was concomitant with SK11 down-regulation. Consistently, immunoblotting with antibody vs. Cyt-G6PDH showed equal protein levels for control and leaves treated with ASO targeted for SK11. This suggested that the silencing of SK11 disrupted the post-translational regulation (by phosphorylation) of Cyt-G6PDH but did not affect the protein synthesis and abundance of the enzyme, as confirmed by immunoblotting. These evaluations allow us to demonstrate the effects of ASO treatment in barley leaves and that HvSK11 modulates the activity of Cyt-G6PDH, using a similar mechanism already identified in *A. thaliana*.

## 4. Materials and Methods

### 4.1. Plant Materials

Seeds of barley (*Hordeum vulgare*, var. *Nure*) were provided by Centro di Ricerca per la Genomica e la Post-genomica Animale e Vegetale (CRA-GPG-Fiorenzuola D’Arda-PC, Piacenza, Italy). Seeds were germinated for 5–7 days in the dark on moistened paper, the seedlings were grown using a hydroponic system for 7 days in darkened plastic bottles, and then they were continuously aerated using an aquarium air pump at 20 °C and 60–80% relative humidity, under a 16 h-light/8 h-dark regime, with approximately 180 μmol photons m^−2^ s^−1^. The composition of the medium Hoagland solution (50 mM KH_3_PO_4_, 50 mM K_2_HPO_4_, 1 mM CaCl_2_, 1.25 mM K_2_SO_4_, 1 mM MgCl_2_, 10 mM KNO_3_, 12.6 μM FeSO_4_—EDTA, 13 μM H_3_BO_3_, 0.24 μM CuSO_4_, 0.35 μM MnSO_4_, 1.5 μM ZnSO_4_, and 0.008 μM (NH_4_)6Mo_7_O_2_) has been previously described [63]. Nutrient solution volume and pH were monitored and adjusted daily. After 7 days of hydroponic growth, plants were divided into three experimental groups: untreated control (CTRL), water-treated, and antisense oligonucleotide (ASO)-treated plants.

*A. thaliana* plants were grown on plates on Murashige and Skoog (MS) medium (Sigma-Aldrich, Saint Louis, MO, USA). Seeds were surface sterilized with ethanol and bleach and then sown on an MS medium and incubated at 4 °C in the dark for 2 days. Plants were then kept in a growth cabinet at 20–22 °C (16 h light/8 h dark photoperiod). After 7 days, plants were transferred into new fresh plates and then grown in 20 cm diameter soil-filled plastic pots at 20 °C, at 60–80% relative humidity, under a 16 h-light/8 h-dark regime, at Photosynthetic Photon Flux Densities (PPFDs) of 150 μmol photons m^−2^ s^−1^ and irrigated regularly. After 1 month, at the full expansion of the rosette, plants were subjected to treatment with ASOs at a concentration of 30 for 6 h.

### 4.2. Preparation and Application of the ASOs

Lyophilized ASOs were dissolved in sterile distilled water to prepare stock solutions, which were stored at 4 °C until use. Working solutions were prepared at final concentrations of 20, 30, or 50 μM. No surfactants or delivery agents were added to the ASO solutions. For each treatment, approximately 300 μL of ASO solution was applied directly to several intact leaves via a soft synthetic brush. The solution was applied drop by drop and gently spread over both the adaxial and abaxial surfaces of the leaves until they were uniformly wetted. Complete contact between ASO solution and leaf tissue was guaranteed during the treatment, minimizing mechanical stress and avoiding visible damage to the tissues. After treatment, the plants were maintained under controlled growth conditions and sampled after 3, 6, or 24 h, depending on the experimental design. Plants treated with an identical volume of sterile distilled water were included as negative controls. Each biological replicate consisted of independently treated plants.

### 4.3. Glucose-6-phosphate Dehydrogenase and 6-Phosphogluconate Dehydrogenase Assays

G6PDH and 6PGDH activities were examined by extracting protein from powdered frozen leaf tissue (250–300 mg) using 600 μL of the extraction buffer containing 50 mM Tris-HCl (pH 8.0), 5 mM MgCl_2_, 4 mM EDTA, 10% (*v*/*v*) glycerol, and 15 μM NADP^+^.

G6PDH activity was assayed according to the method described by Castiglia et al. [64] by monitoring NADP+ reduction at 340 nm. The assay mixture contained: 30 mM Tris–HCl pH 8.0, 5 mM MgCl_2_, 1.5 mM NADP^+^, 30 mM glucose-6P, and the extract. G6PDH assays to determine DTT-sensitive activities were made by incubating samples for 1 h in the presence of dithiothreitol (DTT, Sigma Aldrich, St. Louis, MO, USA) at a final concentration of 100 mM in the dark.

6-phosphogluconate dehydrogenase activity was assayed by monitoring NADP^+^ reduction at 340 nm. The assay mixture was the same as G6PDH, replacing G6P with 3 mM of 6-phosphogluconate. Activities were measured using a Cary 60 spectrophotometer (Agilent Technologies, Santa Clara, CA, USA) equipped with an 18-cell holder and connected to a PC. Activities were expressed as nmol of product min^−1^ mg^−1^ protein.

### 4.4. SDS-PAGE and Immunoblotting

For Western blotting analysis, total proteins were extracted and separated via SDS-PAGE. Leaf tissues were mechanically homogenized using a TissueLyser (Qiagen, Hilden, Germany) at 50 Hz for 2 min, repeated five times, with stainless steel beads (5 mm diameter). Homogenization was performed using a 1:2 (*w*/*v*) ratio of an extraction buffer composed of 50 mM Tris-HCl (pH 7.9), 5 mM MgCl_2_, 4 mM EDTA, and 10% (*v*/*v*) glycerol. The homogenate was centrifuged twice at 13,000 rpm for 20 min and 10 min at 4 °C in a Thermo Scientific SL 16R centrifuge (Thermo Scientific, Waltham, MA, USA), respectively, and the supernatant was used for subsequent analysis. The proteins were then separated by SDS-PAGE and transferred to Comassie blue and/or to a nitrocellulose membrane (Ge Healthcare, Waukesha, WI, USA) using the Transblot-turbo (Biorad, Hercules, CA, USA). Membranes were incubated for 1 h with a primary antibody raised against the cyt-G6PDH (1:20,000) isoform from potato [65] and for 1 h with a secondary antibody anti-rabbit (1:30,000—Merck, Darmstadt, Germany). After incubating the membrane with a horseradish peroxidase (HRP)-linked secondary antibody, cross-reacting polypeptides were identified by enhanced chemiluminescence (ECL) reaction. Densiometric analysis was made using Image-Lab software (version 5.1).

### 4.5. RNA Extraction and qRT-PCR

The RNA was extracted from 100 mg of fresh leaves using a TRIzol reagent (Life Technologies, Carlsbad, CA, USA). The RNA amount was measured using a NanoDrop ND-1000 spectrophotometer (Thermofisher, Waltham, MA, USA). cDNA synthesis was made using the ThermoScript RT-PCR System. The gene expression analysis was carried out by qRT-PCR. Platinum SYBR Green qPCR SuperMix (Life Technologies, Carlsbad, CA, USA). The gene expression was quantified using the 2^−ΔΔCt^ method as in the study by Livak and Schmittgen [66]. The mRNA levels were calculated in each sample, relative to the calibrator. Leaf samples from control plants were used as calibrators; α-tubulin served as endogenous reference genes [62]. Expression values were reported as -fc (fold change—ratio of the normalized expression value; ASO-treated samples vs. CTRL). The list of primers used is in Supplementary Table S1.

### 4.6. Statistical Analysis

Each experiment was performed in at least three biological replicates. Values were expressed as mean ± standard error (SE). The statistical significance of qRT-PCR was evaluated through Student’s *t*-test (*p* ≤ 0.05). The statistical differences in enzymatic activities comparing multiple treatments (CTRL vs. different ASOs/concentrations/time of exposure) were calculated through analysis of variance (ANOVA, α = 0.05). Differences between means were evaluated for significance using the Tukey–Kramer test. Prism v. 6.0 (GraphPad—https://www.graphpad.com) was used for all statistical analyses.

## 5. Conclusions

Our results confirm that the use of ASOs is a reliable and effective strategy for managing enzymes playing central roles in plant metabolism (such as cyt-G6PDH) and also in recalcitrant plant model organisms, such as barley. An ASO-based platform represents a versatile tool for functional genomics in barley and potentially other cereal crops, particularly for the rapid and transient interrogation of gene functions in species that are less amenable to stable transformation. ASOs, in fact, allow us to rapidly test hypotheses about the roles and functions of enzymes involved in primary plant metabolism with no lethal effects or prolonged detrimental consequences. Two barley enzymes (HvCyt-G6PDH and HvSK11) were transiently silenced, showing specific behaviours in ASO-treated leaves: NADPH required in the absence of Cyt-G6PDH and the down-regulation of Cyt-G6PD in SK11-silenced plants. ASO applicability could be extended to the simultaneous targeting of multiple genes, enabling multiplex silencing approaches to dissect complex metabolic and regulatory networks. Treatment with ASO targeted to Cyt-G6PDH induced no evident (or long-term) damage on barley plants, apart from obvious direct effects such as the down-regulation of OPPP enzymes and an increase in the expression of stress-responsive genes. Oligo technology is an alternative to genetically modified organisms because it can specifically modulate the expression of genes rather than altering the genome with unpredictable effects. This strategy offers significant advantages in experimental procedures, including its low cost, high efficiency, and flexibility in adapting to the characteristics of the target plant. Finally, for the first time, our data strongly indicates that SK11 plays an important role in the regulation of Cyt-G6PDH in barley. Future developments should focus on improving ASO delivery efficiency, especially in less accessible tissues such as roots or reproductive organs, and on optimizing stability and uptake under field-relevant conditions. These advancements will be essential to fully exploit ASO technology not only in controlled environments but also for translational applications in crop improvement.

## Data Availability

The original contributions presented in this study are included in the article/Supplementary Materials. Further inquiries can be directed to the corresponding author.

## Supplementary Materials

The following supporting information can be downloaded at https://www.mdpi.com/article/10.3390/plants15142223/s1, Figures S1 and S2: Alignment of the genomic and CDS sequences of different Cyt-G6PDH isoforms from barley; Figure S3. Total G6PDH activity in leaves of *Arabidopsis thaliana* treated with *AtASOcytA* and *HvASOcytA*; Figures S4: Alignment of the CDS sequences of different shaggy kinase isoforms from barley; Figure S5: Coomassie-stained SDS gel of barley leaves from plants treated with HvASOcytA and HvASOsk11A; Table S1: List of primers used for qRT-PCR title.

## Abbreviations

The following abbreviations are used in this manuscript:

G6PDH	Glucose-6-phosphate dehydrogenase
ASO	Antisense oligonucleotide
OPPP	Oxidative pentose phosphate pathway
PGDH	6-phosphogluconate dehydrogenase
SK11	Shaggy-related kinase 11
