# Supplementary material for: Antisense Oligonucleotides as a Gene-Silencing Strategy Regulating Cytosolic G6PDH in Hordeum vulgare
Source: Plants (Basel). 2026 Jul 21;15(14):2223. doi: 10.3390/plants15142223 (PMC13415445; doi:10.3390/plants15142223)

Supplementary Files to

Antisense Oligonucleotides as gene-silencing strategy regulating cytosolic G6PDH in *Hordeum vulgare*

Antonella Aquilone [1-2-3], Maryanna Martina Perrotta [1], Simone Landi [1]* and Sergio Esposito [1]

1 Università di Napoli ''Federico II'', Dipartimento di Biologia, Via Cinthia, I-80126 Napoli, Italy
2 Scuola Superiore Meridionale, Largo San Marcellino 10, 80138 Naples, Italy.
3 Department of Chemical Sciences, University of Napoli Federico II, Naples, Italy.

* Correspondence: Prof Simone Landi - simone.landi@unina.it

Supplementary table

Supplemental Table S1. List of primer used for qRT-PCR

Genes	Sequence
cyt G6PDH Fw	GGCAAGCTGAAGGCTGTTT
cyt G6PDH Rv	GGTCTGCATGTACCCAACCT
P0-G6PDH Fw	ATTTCATCACGTCCCTGGCA
P2-G6PDH Rv	TCGGGTAGGTCACGTAAAACC
α-tub – Fw	CTCCATGATGGCCAAGTGTGA
α-tub – Rv	ATGTCGCTTGGTCTTGATGGT
6PGDH Fw	CAGGAGATCATGGACAGGCA
6PGDH Rv	GGTAGGAGTCGAAGTAGGCC
SK11 Fw	TCAAGATTCTGGGCACACCT
SK11 Rv	TTATGCCATGGATGGGCCTT

Supplementary Figures

Supplemental Figure S1. Alignment of the genomic and CDS sequences of different Cyt-G6PD isoforms from barley (MLOC_13014; MLOC_7841 and MLOC_10808).

```
HvCyt_MLOC_13014_Genomic      ------------------------------------------CCCTTTTGCAAGACCAAG
HvCyt_MLOC_13014_CDS          ------------------------------------------------------------
HvCytMLOC_10808_Genomic       CGTACGCTTCGCGGTTGGTGGCACAGCACAGAGCGCGCAGCCCCCCTTCCCAGGCCGAAA
HvCytMLOC_10808-CDS           ------------------------------------------------------------
HvCyt_MLOC_7841_CDS           ------------------------------------------------------------
HvCyt_MLOC_7841_Genomic       ------------------------------------------------------------

HvCyt_MLOC_13014_Genomic      CTTG--------------------------------------------------------
HvCyt_MLOC_13014_CDS          ------------------------------------------------------------
HvCytMLOC_10808_Genomic       GACGGGTAGGTAGTGGACAGGTGAGGCGAAAGCGGGAGGCGGCCGAGGTGACGCCGTCGT
HvCytMLOC_10808-CDS           ------------------------------------------------------------
HvCyt_MLOC_7841_CDS           ------------------------------------------------------------
HvCyt_MLOC_7841_Genomic       ------------------------------------------------------------

HvCyt_MLOC_13014_Genomic      ------------------------------------------------------------
HvCyt_MLOC_13014_CDS          ------------------------------------------------------------
HvCytMLOC_10808_Genomic       GGTATAAATGGCGGAGCTGCCAATCGCACCCAGTCAGGAAGGAAGGAAGGCGCCACCCCC
HvCytMLOC_10808-CDS           ------------------------------------------------------------
HvCyt_MLOC_7841_CDS           ------------------------------------------------------------
HvCyt_MLOC_7841_Genomic       ------------------------------------------------------------

HvCyt_MLOC_13014_Genomic      ------------------------------------------------------------
HvCyt_MLOC_13014_CDS          ------------------------------------------------------------
HvCytMLOC_10808_Genomic       CTCCCCCCTCCCCACTCCCATCCACCCAGCATCCCAGCAGTAACTCTCCCCCCTCCGCCC
HvCytMLOC_10808-CDS           ------------------------------------------------------------
HvCyt_MLOC_7841_CDS           ------------------------------------------------------------
HvCyt_MLOC_7841_Genomic       ------------------------------------------------------------

HvCyt_MLOC_13014_Genomic      ------------------------------------------------------------
HvCyt_MLOC_13014_CDS          ------------------------------------------------------------
HvCytMLOC_10808_Genomic       CTCCCACGCGGCGCGCGCGCGAATCTCGATCGCCACTGGTGTACTGTTGAAGGGAGAAGA
HvCytMLOC_10808-CDS           ------------------------------------------------------------
HvCyt_MLOC_7841_CDS           ------------------------------------------------------------
HvCyt_MLOC_7841_Genomic       ------------------------------------------------------------

HvCyt_MLOC_13014_Genomic      -----------------ATGATGGTA-------------------CATTTGGAAAGGAGA
HvCyt_MLOC_13014_CDS          -----------------ATGTCAGGAGGATCGGGTGTATCTTCACCATCATTGGGACGAA
HvCytMLOC_10808_Genomic       GAATCATCCTATTCGTCATGGCGGGAAC------TGACTCCTCGGCGTCATCGAGACAAA
HvCytMLOC_10808-CDS           -----------------ATGGCGGGAAC------TGACTCCTCGGCGTCATCGAGACAAA
HvCyt_MLOC_7841_CDS           -----------------ATGGCGGGAAC------TGACTCCTCGGCGTCATCGAGACAAA
HvCyt_MLOC_7841_Genomic       ------------------GACGGAG-----------------------------------
                                                 *  *

HvCyt_MLOC_13014_Genomic      AAAGCATCAGCACATACATCTAAAAATATCGCCTGGAGCATCCTTGAACTGCACACGTAT
HvCyt_MLOC_13014_CDS          TTAGCACGAGTTCTTTGTCAGACTTGCAGGACTTGGAGCTTTCTT---CAGAGTCTGGCT
HvCytMLOC_10808_Genomic       GCAGTTTTAACTCATTAGCAA------AGGATCTAGAACTTCCTT---TGGAGCAAGGGT
HvCytMLOC_10808-CDS           GCAGTTTTAACTCATTAGCAA------AGGATCTAGAACTTCCTT---TGGAGCAAGGGT
HvCyt_MLOC_7841_CDS           GCAGTTTTAACTCATTAGCAA------AGGATCTAGAACTTCCTT---TGGAGCAAGGGT
HvCyt_MLOC_7841_Genomic       ----------------------------------GGAGTATTC--------AGCACAGCT
                                                                 **   * *                *

HvCyt_MLOC_13014_Genomic      CTCTG-CTTTCTTAGAGCTTAGTGCTTTACCAGCCTTAAGGACGAAAGGAACTCCATCCC
HvCyt_MLOC_13014_CDS          GCTTGTCCATTGTTGTACTAGGTGCTTCTGGGGACCT--TGCTAAAAAGAAAACTTTCCC
HvCytMLOC_10808_Genomic       GCCTGACTATCGTTGTACTTGGGGCTTCTGGAGACCT--TGCCAAGAAGAAAACGTTCCC
HvCytMLOC_10808-CDS           GCCTGACTATCGTTGTACTTGGGGCTTCTGGAGACCT--TGCCAAGAAGAAAACGTTCCC
HvCyt_MLOC_7841_CDS           GCCTGACTATCGTTGTACTTGGGGCTTCTGGAGACCT--TGCCAAGAAGAAAACGTTCCC
HvCyt_MLOC_7841_Genomic       ---------TCATTGTATTA----------------------------------------
                                       *  * *   *

HvCyt_MLOC_13014_Genomic      ATCTCTCATTGTGTACCCTCAGCACAACGGATGC--AAAAGTAGGTGTGTTGGATTCATT
HvCyt_MLOC_13014_CDS          GGCACTCTTTA---ACCTTTTTCAACAGGGATTTATACAATCTGGGGAAGTCCATATATT
HvCytMLOC_10808_Genomic       GGCACTCTACC---ACCTTTTTGAACAGGGGTTCTTACAATCTGGTGAAGTGCATATAGT
HvCytMLOC_10808-CDS           GGCACTCTACC---ACCTTTTTGAACAGGGGTTCTTACAATCTGGTGAAGTGCATATAGT
HvCyt_MLOC_7841_CDS           GGCACTCTACC---ACCTTTTTGAACAGGGGTTCTTACAATCTGGTGAAGTGCATATAGT
HvCyt_MLOC_7841_Genomic       --------------ACATTGATGGGCACAGCTTT--------------------------
                                            **  *        *  * *
```

```
HvCyt_MLOC_13014_Genomic      TGGCACTGTTGGATCATCCTTGTAGCCATCATATTGCCCAAGGACTACCTCGT-CATGCT
HvCyt_MLOC_13014_CDS          TGGGTATGCGAGATCAGATATGTC-TGATGACGGGTTAAGAGAACACATTCGTGGATATC
HvCytMLOC_10808_Genomic       TGGGTATGCGAGAACAAATCTTTC-TGATGATGGGTTGAGAGGGCGCATCCGTGCATACC
HvCytMLOC_10808-CDS           TGGGTATGCGAGAACAAATCTTTC-TGATGATGGGTTGAGAGGGCGCATCCGTGCATACC
HvCyt_MLOC_7841_CDS           TGGGTATGCGAGAACAAATCTTTC-TGATGATGGGTTGAGAGGGCGCATCCGTGCATACC
HvCyt_MLOC_7841_Genomic      ----CATGCTGAATCATATATGGA-----------------------TTTGCTAATACA
                                    **     * **     *                            *    **

HvCyt_MLOC_13014_Genomic      TTATAGAGCTCACTGA--------TTGCAGAACCTTGACTTTCTCATCTCGTATGT----
HvCyt_MLOC_13014_CDS          TCAAAGGATCTTCAGATGAACATCTTTCACAATTTTTGCAAT-TGATTAAATATGTTAGT
HvCytMLOC_10808_Genomic       TTAAAGGAGCCTCAGAGGAGCATGTTTCAGAATTCTTGCAAT-TGATAAAATATGTCAGT
HvCytMLOC_10808-CDS           TTAAAGGAGCCTCAGAGGAGCATGTTTCAGAATTCTTGCAAT-TGATAAAATATGTCAGT
HvCyt_MLOC_7841_CDS           TTAAAGGAGCCTCAGAGGAGCATGTTTCAGAATTCTTGCAAT-TGATAAAATATGTCAGT
HvCyt_MLOC_7841_Genomic      TGATAGAACTCT------------TTGTAGAGT--------------------------
                              * * **                  **  * *

HvCyt_MLOC_13014_Genomic      -GCTCAGGACTAAGAGAGACAGGCTTTTCCATGGCTACCAAACAAAAGACCTGCAGCAAA
HvCyt_MLOC_13014_CDS          GGTTCATATGATAATGGGACAGGATTT----GAATTGCTAAACAAGACAATCTCTGAGAA
HvCytMLOC_10808_Genomic       GGCTCCTATGACAGTGGAGAAGGTTTT----GAAAAACTGAACAAGGAAATATCAGATTA
HvCytMLOC_10808-CDS           GGCTCCTATGACAGTGGAGAAGGTTTT----GAAAAACTGAACAAGGAAATATCAGATTA
HvCyt_MLOC_7841_CDS           GGCTCCTATGACAGTGGAGAAGGTTTT----GAAAAACTGAACAAGGAAATATCAGATTA
HvCyt_MLOC_7841_Genomic       ----------------------------------------ACAAA--------------
                                                                       ****

HvCyt_MLOC_13014_Genomic      TGGTTC----------------TGAATAATATCCCGAATGATACCATATTGATCAAAATA
HvCyt_MLOC_13014_CDS          TGAAACATCAGAGAACAACCAACCAGGAAACGCCCGTAGACTATTTTATTTGGCA-----
HvCytMLOC_10808_Genomic       TGAGATGTCAAACAAC------TCAGGAAGCTCCCGTAGGCTCTTTTACTTGGCA-----
HvCytMLOC_10808-CDS           TGAGATGTCAAACAAC------TCAGGAAGCTCCCGTAGGCTCTTTTACTTGGCA-----
HvCyt_MLOC_7841_CDS           TGAGATGTCAAACAAC------TCAGGAAGCTCCCGTAGGCTCTTTTACTTGGCA-----
HvCyt_MLOC_7841_Genomic       -------------------------------------------TGTTCTTAGGCA-----
                                                                              *   *    **

HvCyt_MLOC_13014_Genomic      TCCTCCACGTCCATCAGTTCCAAAGTCCTCCCGGAATACAATCTGTACGTTATCAATGTT
HvCyt_MLOC_13014_CDS          ----TTACCTCCTTCAGTCTACCCCTCAGTATGCAAAATGATAAGATCATATTGCATGAC
HvCytMLOC_10808_Genomic       ----TTGCCTCCATCTGTCTACCCTTCAGTGTGCAAAATGATCCGAACATATTGCATGAG
HvCytMLOC_10808-CDS           ----TTGCCTCCATCTGTCTACCCTTCAGTGTGCAAAATGATCCGAACATATTGCATGAG
HvCyt_MLOC_7841_CDS           ----TTGCCTCCATCTGTCTACCCTTCAGTGTGCAAAATGATCCGAACATATTGCATGAG
HvCyt_MLOC_7841_Genomic       ------------------------------------------------------------

HvCyt_MLOC_13014_Genomic      ATCACGATTCCAGA-GGGGCAGAAAGAAGCGAT--TTGCAAAGC-------GAAGCACCA
HvCyt_MLOC_13014_CDS          TCCATCCTCTCATACTGGTTGGACAAGAGTTATTGTTGAAAAGCCCTTCGGAAAGGATTT
HvCytMLOC_10808_Genomic       TCCAACTTCTCGCACTGGATGGACTAGAGTAATTGTTGAGAAGCCCTTTGGAAGGGACCT
HvCytMLOC_10808-CDS           TCCAACTTCTCGCACTGGATGGACTAGAGTAATTGTTGAGAAGCCCTTTGGAAGGGACCT
HvCyt_MLOC_7841_CDS           TCCAACTTCTCGCACTGGATGGACTAGAGTAATTGTTGAGAAGCCCTTTGGAAGGGACCT
HvCyt_MLOC_7841_Genomic       -----------------------------------TTGAACAGC----------------
                                                                 ***    ***

HvCyt_MLOC_13014_Genomic      GCAAGTTTTGGACTAACTCCTTTCCTAAATAGTGGTCAATTCTATATAGTTGTTCTTCAT
HvCyt_MLOC_13014_CDS          AGACTCTGCTGAGGAATTGAGTGCCCAACTTGGAG------------AGCTTTTCGATGA
HvCytMLOC_10808_Genomic       GGACTCAGCAGAAGAATTAAGTTCCCAACTTGGGG------------AGCTATTCCAGGA
HvCytMLOC_10808-CDS           GGACTCAGCAGAAGAATTAAGTTCCCAACTTGGGG------------AGCTATTCCAGGA
HvCyt_MLOC_7841_CDS           GGACTCAGCAGAAGAATTAAGTACCCAACTTGGGG------------AGCTATTCCAGGA
HvCyt_MLOC_7841_Genomic       ------------------------------------------------------------

HvCyt_MLOC_13014_Genomic      CGAAAAGCTCTCCAAGTTGGGCACTCAATTCCTCAGCAGA-------GTCTAAATCCTTT
HvCyt_MLOC_13014_CDS          AGAACAACTATATAGAATTGA----CCACTATTTAGGAAAGGAGTTAGTCCAAAACTTGC
HvCytMLOC_10808_Genomic       AGATCAACTCTACAGGATTGA----CCACTACTTGGGAAAAGAGTTGGTCCAAAACTTGC
HvCytMLOC_10808-CDS           AGATCAACTCTACAGGATTGA----CCACTACTTGGGAAAAGAGTTGGTCCAAAACTTGC
HvCyt_MLOC_7841_CDS           AGATCAACTCTACAGGATTGA----CCACTACTTGGGAAAAGAGTTGGTCCAAAACTTGC
HvCyt_MLOC_7841_Genomic       -------------------------CCACT------------------------------
                                                        * * *

HvCyt_MLOC_13014_Genomic      CCGAAGGGCTTTTCAACAATAACTCTTGTCCAACCAGTATGAGAGGATGGAGTCATGCAA
HvCyt_MLOC_13014_CDS          TGGTGCTTCGCTTTGCAAATCGCTTCT-TTCTGCCCCTCTGGAATCGTGATAACAT----
HvCytMLOC_10808_Genomic       TTGTGCTTCGTTTTGCGAACCGTTTGT-TCTTACCACTTTGGAACCGTGATAATGT----
HvCytMLOC_10808-CDS           TTGTGCTTCGTTTTGCGAACCGTTTGT-TCTTACCACTTTGGAACCGTGATAATGT----
HvCyt_MLOC_7841_CDS           TTGTGCTTCGTTTTGCGAACCGTTTGT-TCTTACCACTTTGGAACCGTGACAATGT----
HvCyt_MLOC_7841_Genomic      -----------------AATAAATTAT-TGCTACCT-----------------------
                                               **     *  * *     **

HvCyt_MLOC_13014_Genomic      TATGATCTTAT-CATTTTGCATACTGAGGGG-----TAGACTGAAGGAGGTAATGCCAAA
HvCyt_MLOC_13014_CDS          --TGATAACGTACAGATTGTATTCCGGGAGGACTTTGGAACTGATGGACGTGGAG---GA
HvCytMLOC_10808_Genomic   --TGATAATATACAGATTGTATTCAGGGAGGACTTCGGAACTGATGGGCGTGGAG---GA
```

```
HvCytMLOC_10808-CDS           --TGATAATATACAGATTGTATTCAGGGAGGACTTCGGAACTGATGGGCGTGGAG---GA
HvCyt_MLOC_7841_CDS           --TGATAATATACAGATTGTATTCAGGGAGGACTTCGGAACTGATGGGCGTGGAG---GA
HvCyt_MLOC_7841_Genomic       ------------------------------------------------------------


HvCyt_MLOC_13014_Genomic      TAAAATAGTCTACG-GGCGTTTCCTGGTTGGTTGTTCTCTGATGTTTCATTCTCAGAGAT
HvCyt_MLOC_13014_CDS          TATTTTGATCAATATGGTATCATTCGGGATATTATTCAGAACCATTT--GCTGCAGGTCT
HvCytMLOC_10808_Genomic       TATTTTGATCAATATGGAATCATCCGTGATATCATTCAGAACCATTT--GTTGCAGGT--
HvCytMLOC_10808-CDS           TATTTTGATCAATATGGAATCATCCGTGATATCATTCAGAACCATTT--GTTGCAGGTTT
HvCyt_MLOC_7841_CDS           TATTTTGATCAATATGGAATCATCCGTGATATCATTCAGAACCATTT--GTTGCAGGTTT
HvCyt_MLOC_7841_Genomic       -------------------------------------------------TTTGCAGGTTT
                                                                                   ***

HvCyt_MLOC_13014_Genomic      TGTCTTGTTTAGCAATT-CAAATCCTGTCCCATTATCATATGAACCACTAACATATTTAA
HvCyt_MLOC_13014_CDS          TTTGTTTGGTAGCCATGGAAAAGCCTGTCTC-------TCTTAGTCCTGAGCACATA---
HvCytMLOC_10808_Genomic       ------------------AAAAACT-----------------------------------
HvCytMLOC_10808-CDS           TCTGTTTGGTTGCAATGGAAAAGCCTGTATC-------TCTTAAGCCTGAGCACATT---
HvCyt_MLOC_7841_CDS           TCTGTTTGGTTGCAATGGAAAAGCCTGTATC-------TCTTAAGCCTGAGCACATT---
HvCyt_MLOC_7841_Genomic       TCTGTTTGGTTGCAATGGAAAAGCCTGTATC-------TCTTAAGCCTGAGCACATT---
                                                 *** *

HvCyt_MLOC_13014_Genomic      TCAATTGCAAAAATTGTGAAAG---ATGTTC----ATCTGAAGATCCTTTGAGATATCCA
HvCyt_MLOC_13014_CDS          ---------CGAGATGAGAAAGTCAAGGTTCTGCAATCAGTGAGCTCTATAAAG----CA
HvCytMLOC_10808_Genomic       ------------AATAAGA------------------------TTCCATACAA------
HvCytMLOC_10808-CDS           ---------AGAGATGAGAAAGTCAAGGTTCTGCAATCTGTGAACCCGATAAAG----GA
HvCyt_MLOC_7841_CDS           ---------AGAGATGAGAAAGTCAAGGTTCTGCAATCTGTGAACCCGATAAAG----GA
HvCyt_MLOC_7841_Genomic       ---------AGAGATGAGAAAGTCAAGGTTCTGCAATCTGTGAACCCGATAAAG----GA
                                            *  **                            *  *

HvCyt_MLOC_13014_Genomic      CGAATGTGTTCTCTTAACCCGTCATCAG----ACATATCTGATCTCGCATACCCAAATAT
HvCyt_MLOC_13014_CDS          TGACGAGGTAGTCCTTGGGCAATATGATGGCTACAAGGATGATCCAACAGTGCCAAATGA
HvCytMLOC_10808_Genomic       --------TATTATTTCGTCATTTT-----------------------------------
HvCytMLOC_10808-CDS           CGAAGAGGTAGTCCTTGGACAATATCAGGGCTACAAGGATGACCCTACAGTGCCAGATGA
HvCyt_MLOC_7841_CDS           CGAAGAGGTAGTCCTTGGACAATATCAGGGCTACAAGGATGACCCTACAGTGCCAGATGA
HvCyt_MLOC_7841_Genomic       CGAAGAGGTAGTCCTTGGACAATATCAGGGCTACAAGGATGACCCTACAGTGCCAGATGA
                                        *  *  *    *    *

HvCyt_MLOC_13014_Genomic      ATGGACTTCCCCAGATTGTATAAATCCCTGTTGAAAAAGGT---TAAAGAGTGCCGGGAA
HvCyt_MLOC_13014_CDS          ATCCAACACACCTACTTTTGC--ATCCGTTGTGCTGAGGGTACACAATGAGAGATGGGAT
HvCytMLOC_10808_Genomic       -------------ATTATTT--GGCTTTAGTATTTAGTCT---TGATTTGAGACAGTA-
HvCytMLOC_10808-CDS           CTCTAATACCCCAACGTTTGC--ATCTATTGTACTTAGGGTACACAATGAAAGATGGGAA
HvCyt_MLOC_7841_CDS           CTCTAATACCCCAACGTTTGC--ATCTATTGTACTTAGGGTACACAATGAAAGATGGGAA
HvCyt_MLOC_7841_Genomic       CTCTAATACCCCAACGTTTGC--ATCTATTGTACTTAGGGTACACAATGAAAGATGGGAA
                                              * *      *  *  *    *   *     *     *   * *

HvCyt_MLOC_13014_Genomic      AGTTTTCTTTTTAGCAAGGTCCCCAGAAGCACCTAGTACAACAATGGAC-------AAGC
HvCyt_MLOC_13014_CDS          GGAGTTCCTTTCG------TCCTTAAGG----CTGGTAAAGCACTAAGCTCTAAGAAAGC
HvCytMLOC_10808_Genomic       ---------------------------------------AACACTTAAT-----------
HvCytMLOC_10808-CDS           GGTGTCCCTTTCA------TTCTTAAAG----CTGGTAAAGCATTAAACTCAAGAAAAGC
HvCyt_MLOC_7841_CDS           GGTGTCCCTTTCA------TTCTTAAAG----CTGGTAAAGCATTAAACTCAAGAAAAGC
HvCyt_MLOC_7841_Genomic       GGTGTCCCTTTCA------TTCTTAAAG----CTGGTAAAGCATTAAACTCAAGAAAAGC
                                                                     * ** *

HvCyt_MLOC_13014_Genomic      AGCCAGAC-------TCTGAAGAAAGCTCCAAGT----------CCTGCAAGTCTGACAA
HvCyt_MLOC_13014_CDS          AGAGATACGTGTGCAGTTCAAGGATGCTCCAGGCGATATTTTTAGATGCAAG--AAACAA
HvCytMLOC_10808_Genomic       --------------------------TTTTATTTTACTTTTATCCATGTAAG--------
HvCytMLOC_10808-CDS           AGAAATTCGTGTGCAGTTCAAGGATGTTCCCGGTGACATTTTTAAATGTAAG--AAGCAA
HvCyt_MLOC_7841_CDS           AGAAATTCGTGTGCAGTTCAAGGATGTTCCCGGTGACATTTTTAAATGTAAG--AAGCAA
HvCyt_MLOC_7841_Genomic       AGAAATTCGTGTGCAGTTCAAGGATGTTCCCGGTGACATTTTTAAATGTAAG--AAGCAA
                                                         *                  ** ***

HvCyt_MLOC_13014_Genomic      AGAACTCGTGCTAATTCGTCCCAATGATGGTGAAGATACACCCGATCCTCCTGACAT-GG
HvCyt_MLOC_13014_CDS          GGAAGAAATG--AATTTGTC---------------ATACG------CCTGCAGCCATCGG
HvCytMLOC_10808_Genomic       ----------------------------------------------GCTGCGGTTGC---
HvCytMLOC_10808-CDS           GGAAGAAATG--AGTTTGTC---------------ATACG------CCTCCAGCCATCAG
HvCyt_MLOC_7841_CDS           GGAAGAAATG--AGTTTGTC---------------ATACG------CCTCCAGCCATCAG
HvCyt_MLOC_7841_Genomic       GGAAGAAATG--AGTTTGTC---------------ATACG------CCTCCAGCCATCAG
                                                                             ** * *

HvCyt_MLOC_13014_Genomic      TAGTCTAGTCTGTATGGTTTCTCCGTCCTTTCAAGATAACCAG----TGAACACTGAAGG
HvCyt_MLOC_13014_CDS          AAGCCATGTATATGAAATT------AACTGTAAAGAAACCAGGGCTCGAAATGGCAACAG
HvCytMLOC_10808_Genomic       -------------------------CATTGTG----------------------------
HvCytMLOC_10808-CDS           AAGCCATGTATATGAAACT------AACTGTGAAGAAACCTGGATTGGAAATGGCTACTG
HvCyt_MLOC_7841_CDS           AAGCCATGTATATGAAACT------AACTGTGAAGAAACCTGGATTGGAAATGGCTACTG
HvCyt_MLOC_7841_Genomic       AAGCCATGTATATGAAACT------AACTGTGAAGAAACCTGGATTGGAAATGGCTACTG
```

```
                                                          * *

HvCyt_MLOC_13014_Genomic      GACGATGCGGCTTGACTTCGGGAGGAGGGGGGTGGTG----------------CCGGTGG
HvCyt_MLOC_13014_CDS         AACAAAGCGAGCTGGATTTATCCTACGGGCTACGGTACCAAGATATAAAAATCCCAGAAG
HvCytMLOC_10808_Genomic       ------------------------GTGGGTTATGATA--------------TTCCAG---
HvCytMLOC_10808-CDS           AACAGAGTGAACTTGATCTGTCATATGGGATGCGTTACCAAGATGTCAAAATTCCAGAGG
HvCyt_MLOC_7841_CDS           AACAGAGTGAACTTGATCTGTCATATGGGATGCGTTACCAAGATGTCAAAATTCCAGAGG
HvCyt_MLOC_7841_Genomic      AACAGAGTGAACTTGATCTGTCATATGGGATGCGTTACCAAGATGTCAAAATTCCAGAGG
                                                         ***     * *                 ** *

HvCyt_MLOC_13014_Genomic      GATGGGAATATTTTGCTTTG----CTTTGGGTGGAAATTATATATGGCTTTCCTTTCTCG
HvCyt_MLOC_13014_CDS         CATACGAGCGTCTTATCTTGGACACGATAAGAGGTGACCA---GCAGCACTTTGTTCGCC
HvCytMLOC_10808_Genomic       -----------CTTATCC-----------------AACCA--------------------
HvCytMLOC_10808-CDS           CATACGAGCGCCTCATTTTGGATACAATAAGAGGAGACCA---GCAACACTTTGTGCGCC
HvCyt_MLOC_7841_CDS           CATACGAGCGCCTCATTTTGGATACAATAAGAGGAGACCA---GCAACACTTTGTGCGCC
HvCyt_MLOC_7841_Genomic      CATACGAGCGCCTCATTTTGGATACAATAAGAGGAGACCA---GCAACACTTTGTGCGCC
                                         *                        *  *

HvCyt_MLOC_13014_Genomic      CCGCCGCTTTTATACTCGCATCGGCTACTCCCACTCCCCTTTGACCC-------------
HvCyt_MLOC_13014_CDS         GAGACGAGCTGAAGGCTGCCTGGCAGATCTTCACTCCCTTGTTGCACGACATCGATAGTG
HvCytMLOC_10808_Genomic       -------------------------GCCTTTTACCATTTTATT-----------------
HvCytMLOC_10808-CDS           GGGATGAGCTGAAGGCTGCCTGGCAGATCTTCACTCCCTTGTTGCACAACATCGACGCTG
HvCyt_MLOC_7841_CDS           GGGATGAGCTGAAGGCTGCCTGGCAGATCTTCACTCCCTTGTTGCACAACATCGACGCTG
HvCyt_MLOC_7841_Genomic      GGGATGAGCTGAAGGCTGCCTGGCAGATCTTCACTCCCTTGTTGCACAACATCGACGCTG
                                                               **      * *

HvCyt_MLOC_13014_Genomic      GCTGGCTGAACCTTCCACCACATCCACATCTACGCCCTGTTC------------------
HvCyt_MLOC_13014_CDS         GCAGGTTCAAGGCTCTACCATACCAACCGGGAACCCGGGGTCCTCCGGAGGCCGATGAGT
HvCytMLOC_10808_Genomic       ---------AGTGTTTGTTTGACCAACT--------------------------------
HvCytMLOC_10808-CDS           GCAAGCTGAAGGCTGTTTCATACAAGCCTGGCAGCCGTGGCCCCAAGGAAGCTGATGAAC
HvCyt_MLOC_7841_CDS           GCAAGCTGAAGGCTGTTTCATACAAGCCTGGCAGCCGTGGCCCCAAGGAAGCTGATGAAC
HvCyt_MLOC_7841_Genomic      GCAAGCTGAAGGCTGTTTCATACAAGCCTGGCAGCCGTGGCCCCAAGGAAGCTGATGAAC
                                        *    *       *    *

HvCyt_MLOC_13014_Genomic      -----------------TGTTTCTTTTTTCTTTTCCTTTTTCCGGAAAATTGCTGCTTAT
HvCyt_MLOC_13014_CDS         TGAGCAAGAGGACGGGGTACGTGCCGACTCTTGGCTATGTCTGGG-----CACCACCAAC
HvCytMLOC_10808_Genomic       -----------------TATCTCTAGTTCCATGGCTATCTTTTGG-----CAGCAG----
HvCytMLOC_10808-CDS           TGAGTGAGAAGGTTGGGTACATGCAGACCCACGGTTACATCTGGA-----TACCACCCAC
HvCyt_MLOC_7841_CDS           TGAGTGAGAAGGTTGGGTACATGCAGACCCACGGTTACATCTGGA-----TACCACCCAC
HvCyt_MLOC_7841_Genomic      TGAGTGAGAAGGTTGGGTACATGCAGACCCACGGTTACATCTGGA-----TACCACCCAC
                                                *   *       *         *   *

HvCyt_MLOC_13014_Genomic      CCTACCTGGG--------------------------------------------------
HvCyt_MLOC_13014_CDS          CCTTGCAAAG--------------------------------------------------
HvCytMLOC_10808_Genomic       ------------------------------------------------------------
HvCytMLOC_10808-CDS           CCTTGCATAG--------------------------------------------------
HvCyt_MLOC_7841_CDS           CCTTGCATAG--------------------------------------------------
HvCyt_MLOC_7841_Genomic      CCTTGCATAGAGTGCCTCTCTCGATCCTTCTAGGTTGTTTAGCTAGGAAGAGTGAAGGTA

HvCyt_MLOC_13014_Genomic      ------------------------------------------------------------
HvCyt_MLOC_13014_CDS          ------------------------------------------------------------
HvCytMLOC_10808_Genomic       ------------------------------------------------------------
HvCytMLOC_10808-CDS           ------------------------------------------------------------
HvCyt_MLOC_7841_CDS           ------------------------------------------------------------
HvCyt_MLOC_7841_Genomic      TCATGGATAAAAATCTTACGAATTTTCGCTGTACCGTGGTTTACCTGGCGTTGTAATAAC

HvCyt_MLOC_13014_Genomic      ------------------------------------------------------------
HvCyt_MLOC_13014_CDS          ------------------------------------------------------------
HvCytMLOC_10808_Genomic       ------------------------------------------------------------
HvCytMLOC_10808-CDS           ------------------------------------------------------------
HvCyt_MLOC_7841_CDS           ------------------------------------------------------------
HvCyt_MLOC_7841_Genomic      GGATCTGTTTGGCGGAGTATACCTCACCACGGTGTGAAATAAAAAATTTGAGTGTTTCAA

HvCyt_MLOC_13014_Genomic      ------------------------------------------------------------
HvCyt_MLOC_13014_CDS          ------------------------------------------------------------
HvCytMLOC_10808_Genomic       ------------------------------------------------------------
HvCytMLOC_10808-CDS           ------------------------------------------------------------
HvCyt_MLOC_7841_CDS           ------------------------------------------------------------
HvCyt_MLOC_7841_Genomic      TCATTGTTTGTACGACGGTAAGGCGACCTAATATGAGCATCACTTGCTCCTCAGCCTAAG

HvCyt_MLOC_13014_Genomic     --------------------------------------------------------C---
```

```
HvCyt_MLOC_13014_CDS                  --------------------------------------------------------TTTT
HvCytMLOC_10808_Genomic               ------------------------------------------------------------
HvCytMLOC_10808-CDS           ------------------------------------------------------------
HvCyt_MLOC_7841_CDS           ------------------------------------------------------------
HvCyt_MLOC_7841_Genomic  CTAGTCTAAAACGGGCATATTTGTAAGACGTAACTGCTGCCAGGGAGCCTAAGCGTCTAA


HvCyt_MLOC_13014_Genomic      --
HvCyt_MLOC_13014_CDS          AG
HvCytMLOC_10808_Genomic       --
HvCytMLOC_10808-CDS           --
HvCyt_MLOC_7841_CDS           --
HvCyt_MLOC_7841_Genomic       AG
```

Supplemental Figure S2. Alignment of CDS sequences of different Cyt-G6PD isoforms from barley (MLOC_13014; MLOC_7841 and MLOC_10808). Antisense oligo (ASO) sequences are highlighted in blue (*vASOcytA*) and in red (*vASOcyt B*).

```
HvCyt_MLOC_13014_CDS      ATGTCAGGAGGATCGGGTGTATCTTCACCATCATTGGGACGAATTAGCACGAGTTCTTTG
HvCyt_MLOC_7841_CDS       ATGGCGGGA------ACTGACTCCTCGGCGTCATCGAGACAAAGCAGTTTTAACTCATTA
HvCyt_MLOC_10808_CDS      ATGGCGGGA------ACTGACTCCTCGGCGTCATCGAGACAAAGCAGTTTTAACTCATTA
                          *** * ***        **  ** **  * **** * *** **  **    *  ** **

HvCyt_MLOC_13014_CDS      TCAGACTTGCAGGACTTGGAGCTTTCTTCAGAGTCTGGCTGCTTGTCCATTGTTGTACTA
HvCyt_MLOC_7841_CDS       GCAA------AGGATCTAGAACTTCCTTTGGAGCAAGGGTGCCTGACTATCGTTGTACTT
HvCyt_MLOC_10808_CDS      GCAA------AGGATCTAGAACTTCCTTTGGAGCAAGGGTGCCTGACTATCGTTGTACTT
                           **       ****  * ** *** ***  ***   ** *** ** * ** ********

HvCyt_MLOC_13014_CDS      GGTGCTTCTGGGGACCTTGCTAAAAAGAAAACTTTCCCGGCACTCTTTAACCTTTTTCAA
HvCyt_MLOC_7841_CDS       GGGGCTTCTGGAGACCTTGCCAAGAAGAAAACGTTCCCGGCACTCTACCACCTTTTTGAA
HvCyt_MLOC_10808_CDS      GGGGCTTCTGGAGACCTTGCCAAGAAGAAAACGTTCCCGGCACTCTACCACCTTTTTGAA
                          ** ******** ******** ** ******** *************   ******** **

HvCyt_MLOC_13014_CDS      CAGGGATTTATACAATCTGGGGAAGTCCATATATTTGGGTATGCGAGATCAGATATGTCT
HvCyt_MLOC_7841_CDS       CAGGGGTTCTTACAATCTGGTGAAGTGCATATAGTTGGGTATGCGAGAACAAATCTTTCT
HvCyt_MLOC_10808_CDS      CAGGGGTTCTTACAATCTGGTGAAGTGCATATAGTTGGGTATGCGAGAACAAATCTTTCT
                          ***** **  ********** ***** ****** ************** ** ** * ***

HvCyt_MLOC_13014_CDS      GATGACGGGTTAAGAGAACACATTCGTGGATATCTCAAAGGATCTTCAGATGAACATCTT
HvCyt_MLOC_7841_CDS       GATGATGGGTTGAGAGGGCGCATCCGTGCATACCTTAAAGGAGCCTCAGAGGAGCATGTT
HvCyt_MLOC_10808_CDS      GATGATGGGTTGAGAGGGCGCATCCGTGCATACCTTAAAGGAGCCTCAGAGGAGCATGTT
                          ***** ***** ****  * *** **** *** ** ****** * ***** ** *** **

HvCyt_MLOC_13014_CDS      TCACAATTTTTGCAATTGATTAAATATGTTAGTGGTTCATATGATAATGGGACAGGATTT
HvCyt_MLOC_7841_CDS       TCAGAATTCTTGCAATTGATAAAATATGTCAGTGGCTCCTATGACAGTGGAGAAGGTTTT
HvCyt_MLOC_10808_CDS      TCAGAATTCTTGCAATTGATAAAATATGTCAGTGGCTCCTATGACAGTGGAGAAGGTTTT
                          *** **** *********** ******** ***** ** ***** * ***   *** ***

HvCyt_MLOC_13014_CDS      GAATTGCTAAACAAGACAATCTCTGAGAATGAAACATCAGAGAACAACCAACCAGGAAAC
HvCyt_MLOC_7841_CDS       GAAAAACTGAACAAGGAAATATCAGATTATGAGATGTCAAACAAC------TCAGGAAGC
HvCyt_MLOC_10808_CDS      GAAAAACTGAACAAGGAAATATCAGATTATGAGATGTCAAACAAC------TCAGGAAGC
                          ***   ** ******  *** ** **  **** *  *** * ***       ****** *

HvCyt_MLOC_13014_CDS      GCCCGTAGACTATTTTATTTGGCATTACCTCCTTCAGTCTACCCCTCAGTATGCAAAATG
HvCyt_MLOC_7841_CDS       TCCCGTAGGCTCTTTTACTTGGCATTGCCTCCATCTGTCTACCCTTCAGTGTGCAAAATG
HvCyt_MLOC_10808_CDS      TCCCGTAGGCTCTTTTACTTGGCATTGCCTCCATCTGTCTACCCTTCAGTGTGCAAAATG
                           ******* ** ***** ******** ***** ** ******** ***** *********

HvCyt_MLOC_13014_CDS      ATAAGATCATATTGCATGACTCCATCCTCTCATACTGGTTGGACAAGAGTTATTGTTGAA
HvCyt_MLOC_7841_CDS       ATCCGAACATATTGCATGAGTCCAACTTCTCGCACTGGATGGACTAGAGTAATTGTTGAG
Hv_CytMLOC_10808_CDS      ATCCGAACATATTGCATGAGTCCAACTTCTCGCACTGGATGGACTAGAGTAATTGTTGAG
                          **  ** ************ **** * ****  ***** ***** ***** ********

HvCyt_MLOC_13014_CDS      AAGCCCTTCGGAAAGGATTTAGACTCTGCTGAGGAATTGAGTGCCCAACTTGGAGAGCTT
HvCyt_MLOC_7841_CDS       AAGCCCTTTGGAAGGGACCTGGACTCAGCAGAAGAATTAAGTACCCAACTTGGGGAGCTA
HvCyt_MLOC_10808_CDS      AAGCCCTTTGGAAGGGACCTGGACTCAGCAGAAGAATTAAGTTCCCAACTTGGGGAGCTA
                          ******** **** ***  * ***** ** ** ***** *** ********** *****

HvCyt_MLOC_13014_CDS      TTCGATGAAGAACAACTATATAGAATTGACCACTATTTAGGAAAGGAGTTAGTCCAAAAC
HvCyt_MLOC_7841_CDS       TTCCAGGAAGATCAACTCTACAGGATTGACCACTACTTGGGAAAAGAGTTGGTCCAAAAC
HvCyt_MLOC_10808_CDS      TTCCAGGAAGATCAACTCTACAGGATTGACCACTACTTGGGAAAAGAGTTGGTCCAAAAC
                          *** * ***** ***** ** ** *********** ** ***** ***** *********

HvCyt_MLOC_13014_CDS      TTGCTGGTGCTTCGCTTTGCAAATCGCTTCTTTCTGCCCCTCTGGAATCGTGATAACATT
HvCyt_MLOC_7841_CDS       TTGCTTGTGCTTCGTTTTGCGAACCGTTTGTTCTTACCACTTTGGAACCGTGACAATGTT
HvCyt_MLOC_10808_CDS      TTGCTTGTGCTTCGTTTTGCGAACCGTTTGTTCTTACCACTTTGGAACCGTGATAATGTT
                          ***** ******** ***** ** ** ** **  * ** ** ***** ***** **  **

HvCyt_MLOC_13014_CDS      GATAACGTACAGATTGTATTCCGGGAGGACTTTGGAACTGATGGACGTGGAGGATATTTT
HvCyt_MLOC_7841_CDS       GATAATATACAGATTGTATTCAGGGAGGACTTCGGAACTGATGGGCGTGGAGGATATTTT
HvCyt_MLOC_10808_CDS      GATAATATACAGATTGTATTCAGGGAGGACTTCGGAACTGATGGGCGTGGAGGATATTTT
                          *****  ************** ********** *********** ***************
```

```
HvCyt_MLOC_13014_CDS      GATCAATATGGTATCATTCGGGATATTATTCAGAACCATTTGCTGCAGGTCTTTTGTTTG
HvCyt_MLOC_7841_CDS       GATCAATATGGAATCATCCGTGATATCATTCAGAACCATTTGTTGCAGGTTTTCTGTTTG
HvCyt_MLOC_10808_CDS      GATCAATATGGAATCATCCGTGATATCATTCAGAACCATTTGTTGCAGGTTTTCTGTTTG
                          *********** ***** ** ***** *************** ******* ** ******

HvCyt_MLOC_13014_CDS      GTAGCCATGGAAAAGCCTGTCTCTCTTAGTCCTGAGCACATACGAGATGAGAAAGTCAAG
HvCyt_MLOC_7841_CDS       GTTGCAATGGAAAAGCCTGTATCTCTTAAGCCTGAGCACATTAGAGATGAGAAAGTCAAG
HvCyt_MLOC_10808_CDS      GTTGCAATGGAAAAGCCTGTATCTCTTAAGCCTGAGCACATTAGAGATGAGAAAGTCAAG
                          ** ** ************** *******  ***********  *****************

HvCyt_MLOC_13014_CDS      GTTCTGCAATCAGTGAGCTCTATAAAGCATGACGAGGTAGTCCTTGGGCAATATGATGGC
HvCyt_MLOC_7841_CDS       GTTCTGCAATCTGTGAACCCGATAAAGGACGAAGAGGTAGTCCTTGGACAATATCAGGGC
HvCyt_MLOC_10808_CDS      GTTCTGCAATCTGTGAACCCGATAAAGGACGAAGAGGTAGTCCTTGGACAATATCAGGGC
                          *********** **** * * ****** * ** ************** ****** * ***

HvCyt_MLOC_13014_CDS      TACAAGGATGATCCAACAGTGCCAAATGAATCCAACACACCTACTTTTGCATCCGTTGTG
HvCyt_MLOC_7841_CDS       TACAAGGATGACCCTACAGTGCCAGATGACTCTAATACCCCAACGTTTGCATCTATTGTA
HvCyt_MLOC_10808_CDS      TACAAGGATGACCCTACAGTGCCAGATGACTCTAATACCCCAACGTTTGCATCTATTGTA
                          *********** ** ********* **** ** ** ** ** ** ********  ****

HvCyt_MLOC_13014_CDS      CTGAGGGTACACAATGAGAGATGGGATGGAGTTCCTTTCGTCCTTAAGGCTGGTAAAGCA
HvCyt_MLOC_7841_CDS       CTTAGGGTACACAATGAAAGATGGGAAGGTGTCCCTTTCATTCTTAAAGCTGGTAAAGCA
HvCyt_MLOC_10808_CDS      CTTAGGGTACACAATGAAAGATGGGAAGGTGTCCCTTTCATTCTTAAAGCTGGTAAAGCA
                          ** ************** ******** ** ** ****** * ***** ************

HvCyt_MLOC_13014_CDS      CTAAGCTCTAAGAAAGCAGAGATACGTGTGCAGTTCAAGGATGCTCCAGGCGATATTTTT
HvCyt_MLOC_7841_CDS       TTAAACTCAAGAAAAGCAGAAATTCGTGTGCAGTTCAAGGATGTTCCCGGTGACATTTTT
HvCyt_MLOC_10808_CDS      TTAAACTCAAGAAAAGCAGAAATTCGTGTGCAGTTCAAGGATGTTCCCGGTGACATTTTT
                           *** *** *  ******** ** ******************* *** ** ** ******

HvCyt_MLOC_13014_CDS      AGATGCAAGAAACAAGGAAGAAATGAATTTGTCATACGCCTGCAGCCATCGGAAGCCATG
HvCyt_MLOC_7841_CDS       AAATGTAAGAAGCAAGGAAGAAATGAGTTTGTCATACGCCTCCAGCCATCAGAAGCCATG
HvCyt_MLOC_10808_CDS      AAATGTAAGAAGCAAGGAAGAAATGAGTTTGTCATACGCCTCCAGCCATCAGAAGCCATG
                          * *** ***** ************** ************** ******** *********

HvCyt_MLOC_13014_CDS      TATATGAAATTAACTGTAAAGAAACCAGGGCTCGAAATGGCAACAGAACAAAGCGAGCTG
HvCyt_MLOC_7841_CDS       TATATGAAACTAACTGTGAAGAAACCTGGATTGGAAATGGCTACTGAACAGAGTGAACTT
HvCyt_MLOC_10808_CDS      TATATGAAACTAACTGTGAAGAAACCTGGATTGGAAATGGCTACTGAACAGAGTGAACTT
                          ********* ******* ******** **   * ******** ** ***** ** ** **

HvCyt_MLOC_13014_CDS      GATTTATCCTACGGGCTACGGTACCAAGATATAAAAATCCCAGAAGCATACGAGCGTCTT
HvCyt_MLOC_7841_CDS       GATCTGTCATATGGGATGCGTTACCAAGATGTCAAAATTCCAGAGGCATACGAGCGCCTC
HvCyt_MLOC_10808_CDS      GATCTGTCATATGGGATGCGTTACCAAGATGTCAAAATTCCAGAGGCATACGAGCGCCTC
                          *** * ** ** *** * ** ********* * ***** ***** *********** **

HvCyt_MLOC_13014_CDS      ATCTTGGACACGATAAGAGGTGACCAGCAGCACTTTGTTCGCCGAGACGAGCTGAAGGCT
HvCyt_MLOC_7841_CDS       ATTTTGGATACAATAAGAGGAGACCAGCAACACTTTGTGCGCCGGGATGAGCTGAAGGCT
HvCyt_MLOC_10808_CDS      ATTTTGGATACAATAAGAGGAGACCAGCAACACTTTGTGCGCCGGGATGAGCTGAAGGCT
                          ** ***** ** ******** ******** ******** ***** ** ************

HvCyt_MLOC_13014_CDS      GCCTGGCAGATCTTCACTCCCTTGTTGCACGACATCGATAGTGGCAGGTTCAAGGCTCTA
HvCyt_MLOC_7841_CDS       GCCTGGCAGATCTTCACTCCCTTGTTGCACAACATCGACGCTGGCAAGCTGAAGGCTGTT
HvCyt_MLOC_10808_CDS      GCCTGGCAGATCTTCACTCCCTTGTTGCACAACATCGACGCTGGCAAGCTGAAGGCTGTT
                          ****************************** *******   ***** * * ****** *

HvCyt_MLOC_13014_CDS      CCATACCAACCGGGAACCCGGGGTCCTCCGGAGGCCGATGAGTTGAGCAAGAGGACGGGG
HvCyt_MLOC_7841_CDS       TCATACAAGCCTGGCAGCCGTGGCCCCAAGGAAGCTGATGAACTGAGTGAGAAGGTTGGG
HvCyt_MLOC_10808_CDS      TCATACAAGCCTGGCAGCCGTGGCCCCAAGGAAGCTGATGAACTGAGTGAGAAGGTTGGG
                           ***** * ** ** * *** ** **    *** ** *****  ****   *** *   ***

HvCyt_MLOC_13014_CDS      TACGTGCCGACTCTTGGCTATGTCTGGGCACCACCAACCCTTGCAAAGTTTTAG
HvCyt_MLOC_7841_CDS       TACATGCAGACCCACGGTTACATCTGGATACCACCCACCCTTGCA------TAG
HvCyt_MLOC_10808_CDS      TACATGCAGACCCACGGTTACATCTGGATACCACCCACCCTTGCA------TAG
                          *** *** *** *  ** **  *****  ****** *********      ***
```

Supplemental Figure S3. Total G6PD activity in leaves of *Arabidopsis thaliana* under control conditions (CTRL - black bar) and treated with *AtASOcytA* (dark grey bar) and *vASOcytA* (light grey bar) at 30 μM for 6h. Letters indicate the statistical significance between control and treated plants using ANOVA.

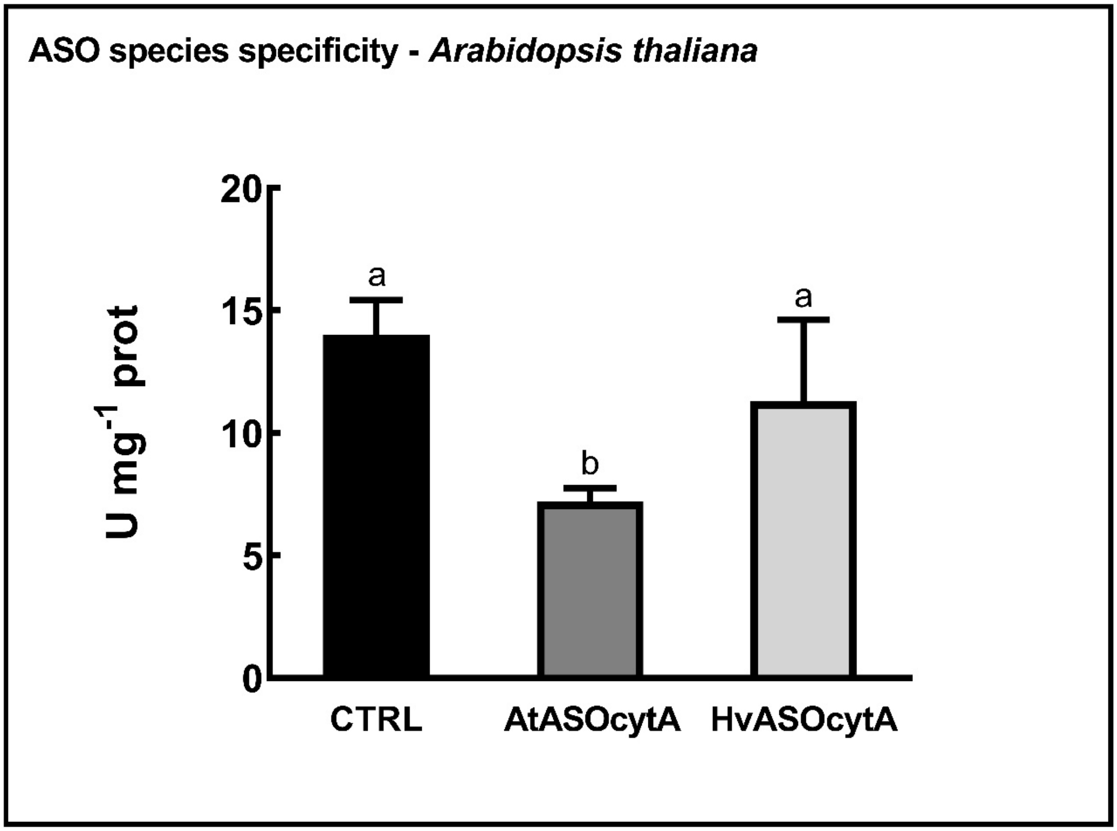

Supplemental Figure S4. Alignment of CDS sequences of different SAGGY -like Kinases/ glycogen synthase kinases (GSKs) isoforms from barley (ORVU3r1G034440; ORVU3r1G034440 and ORVU1r1G016490). Antisense oligo (ASO) sequences are highlighted purple (*vASOsk11A*) and in in green (*vASOsk11B*).

```
HORVU5Hr1G117030      -----CTCCCTCTCATTTGCTCTGCTCGCTCGCTCGCTCGCTCGGCGCCCCCTCCTCTCC
HORVU3Hr1G034440      CGCGTACCCGATCCACGACCCCGACACGCGGGGCCCGGAAGCTGAATGGCCCACCTGTAC
HORVU1Hr1G016490      -----GACCACCACACCACAGCGACGCGC------------------CCCCCGCACCCGC
                             **    **      *  * ***                    *** *     *

HORVU5Hr1G117030      TCCGATCCTCCCCCCACGTCCCGCGCCGGAGCTGGGGAGGGAGGGGGGAAATCCATC---
HORVU3Hr1G034440      GTGGG---------CGCATAGGGGAGCGGGGCCGTGCCGGGCGACGCGCGTTCCCTTGGT
HORVU1Hr1G016490      TGCGA---------------CTGGACTGGACTGGACTGGAGTGGAGCGCACTCCAGTGGA
                         *                  *    **    *    * * *  * *   ***

HORVU5Hr1G117030      -----------------------------CCCGCGTC--------------AGGCGGATT
HORVU3Hr1G034440      GGTGG--------TGGGTTGCTTTGACTGCCCTCGGCAGCAGGCGGGGCGCAGGCACAAA
HORVU1Hr1G016490      GACAGCTGCGGACCAGGCCCTCCGATCCGCCCGCGCC--CACGCCCGCCGCAGCCCCCTA
                                                   *** ** *              ** *

HORVU5Hr1G117030      CCGTCCCGGATCC--CCGCGTCGGGCACGCCGGCCT------------------------
HORVU3Hr1G034440      TCATTTCGGCCGCTGCGTCGCCCGTCCCTCCGCCTTGGCCTTGGCACGGTGGTGGTAGAG
HORVU1Hr1G016490      TGGTCCCGCCGCCCGCCGGACC-GCCTCGCCGCCGC------------------------
                         *  **    *  *     * * * * *** *

HORVU5Hr1G117030      ------------------------------------------------------------
HORVU3Hr1G034440      TAGGAGTAGTAGTAGTAGTACTACTATCTCAGTTGCGTCCCGACAACCAAAAGGAGAGAG
HORVU1Hr1G016490      ------------------------------------------------------------


HORVU5Hr1G117030      -------------------GCTAACCCGGTTA----------------------------
HORVU3Hr1G034440      CGGAGAGAGGAAAGGGGAAGGCAACCCAGCTGCAACACAGGGCGGAGCGAGCGCGGGGTA
HORVU1Hr1G016490      -------------------ACCAACCCGGTAATAACA-----------------------
                                            ***** *

HORVU5Hr1G117030      -----------------------ACCAAGTAAGGTGCATTCTGAA----CGGTAGCCTGT
HORVU3Hr1G034440      GGGGAGAATCGACGGATCTCTGTGTCAAGGAGGAGATACACCAAAACACCTGTCGCTTCT
HORVU1Hr1G016490      ----------------------TGTTCAG-------TGCTCTGAA--------AGTTTCA
                                                 **           *  **         *  *

HORVU5Hr1G117030      AAAACCTTATT---AG---------------GCAATTCTACCACATTATGGCCTCGGTTG
HORVU3Hr1G034440      GTGATTTGATGCGCAGCTGAATTGGTGTCTCTTGGCTTCTGTAAGAGATGGCTTCTGTAG
HORVU1Hr1G016490      GAGTCATAATTCCAAG---------------TCAGCTC--------AATGACCTCATTTG
                            * **    **                    *          *** * **  * *

HORVU5Hr1G117030      GTGCGGTGCGTCCTTCCTCGCGCTTTCAGAATGACACGAGTACTAGTGGTGATGCCGAAC
HORVU3Hr1G034440      GCGTTGCTC---CGTCCGGGCAC---AAGAACAGCAGCGGCACCAGCATGGGTGTTGAGA
HORVU1Hr1G016490      GTGTCGCACCGGCATCTGGGCT------GAGAGATGCTGGTGGTAGTAGTGAAGTAGATA
                      * *  *  *   * **   **       **         *    **    *  *  **

HORVU5Hr1G117030      GACTTCCGAACGAGATGGGCAATATGAGCATAAGGGATGACAGGGACCCTGAAGATATAG
HORVU3Hr1G034440      AGCTACCAGATCAGATGAACGATTTGAAGATCAGAGATGATAAGGAAGTGGAAGCTACCA
HORVU1Hr1G016490      GATTGCCGGATGAAATTAGTAATATGAGGATAAGTGATGAAAAGGAAGTAGAGGCAACAA
                         * **  *  * **     ** ***  ** ** ***** * ***    ** *  *

HORVU5Hr1G117030      TAGTCAACGGCAATGGGACGGAACCAGGCCATATTATAGTCACAAGCATTGAGGGAAGAA
HORVU3Hr1G034440      TTATTAATGGTAAGGGAACAGAGACTGGTCACATAATTGTCACGACTACTGGTGGCAAAA
HORVU1Hr1G016490      TCATCAATGGGAATGGAACAGAAGCTGGTCATATTATAGTCACAACTATCGGAGGCAGAG
                      *  * ** ** ** ** ** **  * ** ** ** ** ***** *  *  *  ** * *

HORVU5Hr1G117030      ATGGGCAAGCAAAACAGACCATTAGCTACATGGCTGAGCGCGTGGTTGGTCATGGGTCAT
HORVU3Hr1G034440      ATGGTCAGCCGAAACAGACCGTGAGCTACATGGCTGAGCGGATTGTTGGTCAAGGTTCAT
HORVU1Hr1G016490      ATGGCCAAAGGAAGCAGACAATAAGTTACATGGCTGAACGTGTCATTGGTCAAGGATCAT
                      **** **    ** *****  * ** *********** **  *  ******* ** ****

HORVU5Hr1G117030      TTGGAACTGTTTTCCAGGCCAAGTGTCTTGAAACTGGCGAGACGGTGGCTATAAAGAAGG
HORVU3Hr1G034440      TTGGTATTGTATTCCAGGCTAAATGTTTGGAGACAGGGGAAACTGTTGCCATTAAGAAGG
HORVU1Hr1G016490      TTGGTGTTGTGTTCCAGGCAAAATGTTTGGAGACAAGTGAGACAGTAGCTATCAAGAAGG
                      ****   *** ******** ** *** * ** **  * ** ** ** ** ** *******
```

```
HORVU5Hr1G117030    TTCTTCAAGACAAGAGATATAAGAACCGTGAGCTGCAAACGATGCGAGTTCTTGACCATC
HORVU3Hr1G034440    TCCTTCAGGACAAGCGCTACAAGAACCGTGAGCTACAGACCATGCGCCTGCTTGACCACC
HORVU1Hr1G016490    TCCTTCAGGATAAGAGATACAAGAACCGTGAGTTGCAAATGATGCGTCTACTTGACCATC
                    * ***** ** *** * ** ************ * ** *  *****  * ******** *

HORVU5Hr1G117030    CAAATGTTGTGGCTTTGAAGCATTGTTTTTTCTCGAAGACTGAGAAGGAGGAGCTTTACC
HORVU3Hr1G034440    CAAATGTCGTAGCTCTGAAGCATTGCTTCTTTTCTACAACTGAGAAGGATGAACTGTATC
HORVU1Hr1G016490    CAAATGTCGTCTCTCTGAAACACTGCTTCTTCTCAACCACTGAAAAGGATGAACTCTTTC
                    ******* **  ** **** ** ** ** ** ** *  ***** ***** ** ** *  *

HORVU5Hr1G117030    TCAACCTGGTGCTTGAATATGTGCCAGAGACTGCTCATCGTGTTATTAAGCATTACAACA
HORVU3Hr1G034440    TAAACTTGGTTCTCGAGTATGTGCCTGAGACTGTCCATCGTGTTGTCAAGCATTACAACA
HORVU1Hr1G016490    TGAATCTTGTGCTTGAGTATGTTCCTGAGACAGTCCACCGTGTCATCAGACACTACAACA
                    * **  * ** ** ** ***** ** ***** *  ** *****  * *  ** *******

HORVU5Hr1G117030    AGATGAACCAACGCATGCCCTTGATATATGCAAAACTGTACATGTATCAGATATGTAGAT
HORVU3Hr1G034440    AGATGAACCAGCGCATGCCACTAATCTATGTGAAGCTGTATACATACCAGATATGTAGGG
HORVU1Hr1G016490    AGATGAACCAACGCATGCCACTGATATATGTGAAACTATATTCTTATCAGATTTGTAGGG
                    ********** ********  * ** ****  ** ** **    ** ***** *****

HORVU5Hr1G117030    CTTTGGCATACATTCACAACAGCATTGGAGTATGTCACAGAGACATCAAGCCTCAAAATC
HORVU3Hr1G034440    CTCTGGCTTACATTCATGGCAGCATTGGAGTTTGCCACAGGGATATCAAGCCACAAAATC
HORVU1Hr1G016490    CACTGGCTTATATCCATCGCACTGTTGGTGTTTGCCACAGGGACATAAAACCACAGAATC
                    *  **** ** ** **   **   **** ** ** ***** ** ** ** ** ** ****

HORVU5Hr1G117030    TTCTGGTGAATCCACATACTCATCAGTTGAAATTATGTGACTTTGGAAGCGCGAAAGTGT
HORVU3Hr1G034440    TTCTGGTAAACCCACATACCCACCAGCTGAAACTGTGTGACTTTGGGAGTGCAAAAGTTC
HORVU1Hr1G016490    TTCTGGTTAACCCACACACTCATCAGCTGAAGATCTGTGATTTTGGAAGTGCAAAAGTTC
                    ******* ** ***** ** ** *** ****  * ***** ***** ** ** *****

HORVU5Hr1G117030    TGGTGAAAGGAGAGCCAAATATTTCCTATATCTGTTCAAGGTACTATAGAGCCCCAGAGC
HORVU3Hr1G034440    TTGTTAAGGGAGAACCAAACATATCATATATTTGTTCCCGATACTATAGGGCTCCAGAGC
HORVU1Hr1G016490    TGGTGAAAGGCGAACCCAATATATCATACATCTGCTCTAGGTACTATAGGGCACCAGAGC
                    * ** ** ** ** ** ** ** ** ** ** ** ** **  * ******** ** *******

HORVU5Hr1G117030    TCATATTTGGTGCTACTGAATACACAACGGCAATTGATGTTTGGTCTGCTGGCTGTGTTC
HORVU3Hr1G034440    TCATATTTGGTGCTACTGAGTACACTACGGCCATTGACATTTGGTCTGCTGGATGTGTTC
HORVU1Hr1G016490    TTATTTTTGGTGCTACTGAGTACACAACAGCAATCGACATCTGGTCTGCTGGATGTGTTC
                    * ** ************** ***** ** ** ** **  * *********** *******

HORVU5Hr1G117030    TTGCTGAACTCCTTCTAGGACAGCCTATATTCCCTGGCGACAGTGGTGTTGATCAGCTTG
HORVU3Hr1G034440    TTGCTGAGCTTATGCTAGGGCAGCCTCTGTTTCCTGGTGAAAGTGGTGTGGACCAGCTTG
HORVU1Hr1G016490    TTGCTGAACTTCTGACAGGACAGCCTCTGTTTCCTGGTGAAAGTGGAGTTGATCAACTTG
                    ******* **  *   *** ****** * ** ***** ** ***** ** ** ** ****

HORVU5Hr1G117030    TTGAAATCATCAAGGTTTTAGGTACCCCTACACGAGAAGAAATTAAGTGCATGAATCCAA
HORVU3Hr1G034440    TTGAAATCATCAAGGTCCTTGGTACCCCTACAAGGGAAGAAATTAAATGCATGAACCCAA
HORVU1Hr1G016490    TTGAAATTATCAAGATTCTGGGCACACCTACAAGGGAAGAAATCAAATGTATGAACCCGA
                    ******* ****** *  * ** ** ****** * ******** ** ** ***** ** *

HORVU5Hr1G117030    ATTATACCGAGTTTAAATTCCCGCAAATCAAAGCTCACCCATGGCACAAGATCTTCCATA
HORVU3Hr1G034440    ATTATACGGAGTTCAAATTCCCACAGATTAAAGCACACCCTTGGCACAAGGTATTCCATA
HORVU1Hr1G016490    ACTACACAGAATTCAAGTTTCCCCAAATCAAGGCCCATCCATGGCATAAGGTATTCCACA
                    * ** ** ** ** ** ** ** ** ** ** ** ** ** ***** *** * ***** *

HORVU5Hr1G117030    AAAGAATGCCTGCTGAAGCAGTAGATCTTGTCTCGAGACTCTTGCAATATTCACCAAGCC
HORVU3Hr1G034440    AAAGGATGCCACCTGAAGCTGTTGATCTGGTCTCTCGGCTCCTCCAGTACTCGCCCAACC
HORVU1Hr1G016490    AGCGAATGCCGCCTGAAGCAGTGGACCTGGTCTCCAGGCTTCTTCAGTACTCACCGCACC
                    *  * *****  ******* ** ** ** *****  * **  * ** ** ** **   **

HORVU5Hr1G117030    TGCGTTCGACTGCTTTGGAAGCATTAATTCATCCATTCTTCGATGAACTCCGGGACCCAA
HORVU3Hr1G034440    TAAGATGCACTGCTGTGGAGGCACTTGTTCACCCCTTCTTTGATGAGCTTCGGGACCCGG
HORVU1Hr1G016490    TTCGGTCCTCAGCTCTGGACGCCCTGATCCATCCGTTCTTCGACGAGCTCCGGGACCCAA
                    *  * *   * *** **** **  *  * ** ** ***** ** ** ** ********

HORVU5Hr1G117030    ACACCCGCTTACCGAATGGCCGTTTTCTTCCTCCCCTCTTTAACTTCAAGCCCCATGAGT
HORVU3Hr1G034440    GTACCCGCCTTCCGAATGGTCGCTTTCTGCCTCCTCTTTTTAACTTCAAGCCTAACGAAC
HORVU1Hr1G016490    ACACACGCCTCCCCAACGGCCGGTTCCTACCTCCCCTCTTCAACTTCAAGCCTCACGAGC
                      ** *** * ** ** ** ** ** ** ** **** ** ** *********** **  * **
```

```
HORVU5Hr1G117030      TGAAGGGCGTGCCGATGGACATCCTGGTGAAGCTCATCCCTGAACATGCTCGGAAGAACT
HORVU3Hr1G034440      TGAAAGGAATCCCAGTGGATGTTGCGGCAAAACTGATCCCGGAGCACGCGAGAAAGCAGA
HORVU1Hr1G016490      TGAAGGGGCTTCCAATGGAGATTGCAATGAAGCTGGTCCCCGAGCACGCGAGGAGCCAAT
                      **** **  * **  ****  *       ** **  **** ** ** **  * *   *

HORVU5Hr1G117030      GCGCCTTTGTAGGGTGGTG----------ATCCGACGGCTGTTGAAGCTTAGTTCA----
HORVU3Hr1G034440      GCTCCCATGCATGACTATGAGATGCCTGTGTCAGAT--TCGTTGGAATTGCATCGACAAG
HORVU1Hr1G016490      GCCCCTTCCTAGGGCTGTAGG--------GTCTGTC--TTGTTG--GCTACAT-------
                      ** **     * *    *            ** *      ****    *   *

HORVU5Hr1G117030      -GAACAAATCCTATTGTC--GTCTACTAGAAACCCCAGGAGTTTGA--GATTGCCTGCAG
HORVU3Hr1G034440      TGAGCATTTCCCCTGGGGATGTTGGTTGGGCAGCATGCGAGCCTGATGAACTACCTATGG
HORVU1Hr1G016490      -GTGCATTGCTGTCTGTCATGCTAGC-GAATAGCGC-----TCCTACGTCCTACCTAGAG
                       *  **   *     *    *          * *           *     * ***   *

HORVU5Hr1G117030      C---TACATGGGATATAGGCGATGCA----CATGTGATTATTATTCTTTTTCTTGTCCGA
HORVU3Hr1G034440      C-----TGTTGGA-AGACGTGATGTATGACCAC----------TCCACCCTGTTGACCTG
HORVU1Hr1G016490      ACCAGGCGTTGGA-ACAACTGA-ACATGATCATGTGGGTGGTGTCTTTGCTGTTGCTTTG
                              * *** * *   **   *    **           *      * ***

HORVU5Hr1G117030      GAC--CTCGGTGTCATGTATTCTTTCTTTCCCCCTACTGCCGATGTAA-CAAATCACCCA
HORVU3Hr1G034440      TGTAGCTGAGTAGTAAGTATGCTGCGATGCAAGCT--TGACCATGTAACCGGTTCACCCT
HORVU1Hr1G016490      GACGGTT------TGCGTGTGGTCTTGAACTGCCA--GGACAAAGAGTCCGAATGACC--
                            *         ** *  *      *   *    * * * *    *   * ***

HORVU5Hr1G117030      TGATTCTGTAA----------GTAGATGAGAAGTGTTTCGACTGTTCTCCCCTGAGCTC-
HORVU3Hr1G034440      CCCTTGTACTAACCCTCGCCTGAGAATTGAGATTGCCATAGCTGATCTGTAATATGTTCT
HORVU1Hr1G016490      ---------GA----------GTAAGTGAGGATTGCAGTAACAGAAACGCAGTAA-TTAT
                                *          *    *    * **      * *        *    *

HORVU5Hr1G117030      ------------ATGTGCTATGCAAGGAAGGATGCACAC-TATGTACCGCCAATATTTGG
HORVU3Hr1G034440      AGAGAAGTATGAATGTATT-TATTGTAGTTGATCCTTGTATGGGTCCTCTCGATTTT---
HORVU1Hr1G016490      GGCTATGCTTTGATGAATTATGTTGAAGACGATGATGATGTCGGTGCCATCGACTCTTGT
                                  ***   * *         ***       *  ** *   * *   *

HORVU5Hr1G117030      TCCAGT------ATTTGTTCATGGATCGAGGCTATAATTCATTTGTACTCATCACATTGT
HORVU3Hr1G034440      CCTAGT---------------------------------------TTCTTAATTGTCAGA
HORVU1Hr1G016490      CCGAGTTAGAATGGGCGTCCAAAAAGTTATGAGATGGCATGAAAATATTCAACGAACAGA
                       * ***                                       *  * *       *

HORVU5Hr1G117030      CTTGTCAGTTACTGTCAAGTTTTTCAATTGGAC
HORVU3Hr1G034440      CTCGT----------------------------
HORVU1Hr1G016490      GTGATT-GCGTCCATCATATGGTGAAATTTCG-
                       *  *
```

Supplemental Figure S5. Coomassie-stained SDS gel of barley leaves from plants grown under control conditions (CTRL) and treated with 30 µM *vASOcytA* and *vASOsk11A* for 6h.

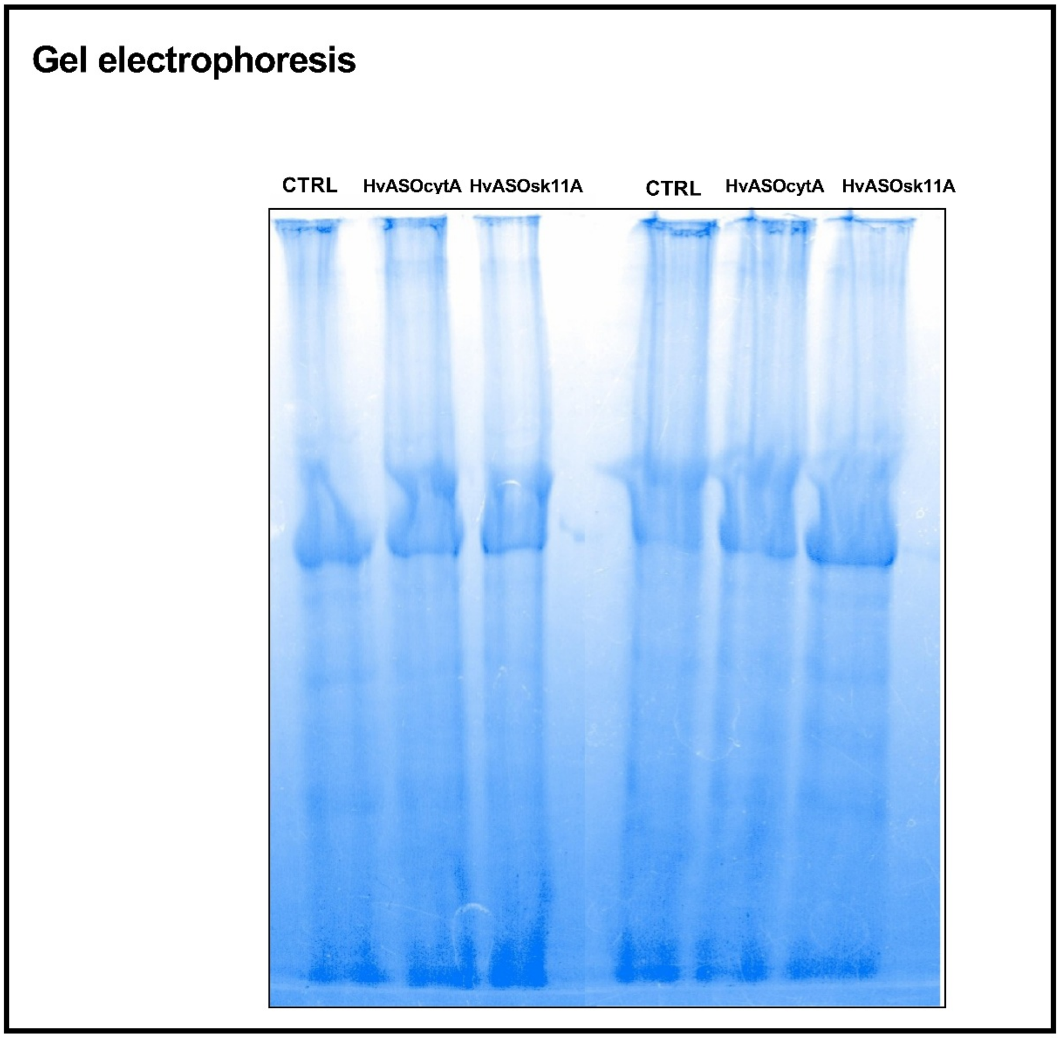

Supplement: Supplementary file 1 [file plants-15-02223-s001.zip › plants-4422138-supplementary.pdf]
